# Peptide ligands to explore interactions with intrinsically disordered multidomain proteins: the case of SARS-CoV-2 nucleocapsid protein

**DOI:** 10.1038/s41598-026-46442-9

**Published:** 2026-04-18

**Authors:** A. S. Tino, M. Quagliata, M. Schiavina, L. Attanasio, B. P. O. Santos, L. Pacini, A. M. Papini, R. Pierattelli, I. C. Felli

**Affiliations:** 1 Department of Chemistry “Ugo Schiff”, University of Florence, Via Della Lastruccia 3-13, 50019 Sesto Fiorentino, Florence, Italy; 2 Magnetic Resonance Center (CERM), University of Florence, Via Luigi Sacconi 6, 50019 Sesto Fiorentino, Florence, Italy; 3 Interdepartmental Research Unit of Peptide and Protein Chemistry and Biology (PeptLab), University of Florence, Via Della Lastruccia 13, 50019 Sesto Fiorentino, Florence, Italy

**Keywords:** SARS-CoV-2 nucleocapsid protein, Intrinsically disordered protein regions (IDRs), Synthetic tailored peptides, Peptide-protein interactions, ^19^F NMR spectroscopy, Molecular dynamics simulations, Biochemistry, Biophysics, Chemical biology, Chemistry, Structural biology

## Abstract

The SARS-CoV-2 nucleocapsid protein (N), essential for viral RNA packaging, comprises a structured RNA-binding domain, NTD(44–180), flanked by intrinsically disordered regions, IDR1 (1–43) and IDR2 (181–248). This extended, positively charged RNA-binding surface makes it an attractive target for synthetic ligands able to interfere with its function. In this study, peptide ligands were explored as synthetically accessible alternatives for modulating N interactions. After confirming the ability of a reference peptide (P0) in engaging IDRs in the interaction, we shifted our attention to key properties that modulate the interaction with the central domain NTD(44–180). Two new peptides (P1 and P2) were designed with identical amino acid compositions but distinct sequence arrangements. In each one a para-fluorinated phenylalanine residue was incorporated to introduce an aromatic component useful to mimic RNA bases and enable ^19^F NMR detection. Molecular dynamics simulations, circular dichroism, and NMR spectroscopy were combined to characterize the structural, dynamic and binding properties of P1 and P2. Both peptides involve the RNA-binding groove of the N protein (NTD(44–180)). While P1 demonstrated higher α-helical propensity and favorable electrostatic interactions, P2 displayed stronger interactions through enhanced conformational adaptability. ^19^F NMR experiments confirmed complex formation also from the ligand perspective. These findings support a modular peptide design strategy for targeting multidomain proteins.

**Supplementary Information:**

The online version contains supplementary material available at 10.1038/s41598-026-46442-9.

## Introduction

The SARS-CoV-2 nucleocapsid (N) protein interacts with RNA to accomplish various key functions for the virus life cycle, such as genome packing, transcription and replication of viral RNA, and assembly of new virions in the cell^1–4^. A major role in this interaction is played by the highly flexible regions of the N-terminal globular domain [NTD(44–180)]^5–8^, and by its flanking intrinsically disordered regions [IDR1(1–43) and IDR2(181–248)]^9–14^. Given its critical role in the virus life cycle, this interaction is a promising target for the rational design of novel inhibitors. However, the protein exhibits extensive disorder and flexibility, a key functional property that prevents a well-defined three-dimensional structure characterization, thereby limiting the use of mainstream approaches in new inhibitors’ discovery.

The presence of highly dynamic polypeptide regions in proteins over the years have been shown to be very important in modulating a variety of biological mechanisms^15–17^ and are often involved in the insurgence of a wide number of pathologies^18^. The absence of a rigid template to rely on, provided by the presence of a well-defined fold as for more common protein–protein or protein-nucleic acids interactions, is particularly challenging.

Recent work focused on the investigation of linear polyanions as possible ligands of N targeting the highly flexible, positively charged regions present in NTD(44–180) and in its flanking disordered regions (IDR1(1–43) and IDR2(181–248)) that are part of NTR(1–248)^19–21^. Indeed IDR1(1–43) and IDR2(181–248) as well as flexible regions of NTD(44–180) were found to play an important role in modulating interactions with RNA^6,10,12,22–24^ and potential ligands^19–21^. These regions are characterized by high flexibility, low sequence complexity, and prevalence of polar and charged residues, which contribute to their ability to establish transient interactions and undergo dynamic conformational changes. Their presence enhances the adaptability of the NTD(44–180) to various binding partners and may influence ligand binding by providing additional contact points or by modulating the accessibility of the structured core.

Previous work focused on enoxaparin^19,25^, one of the most negatively charged natural compounds, and on smaller enoxaparin fragments^21^. As an alternative route, a chimaera was designed with a central core constituted by peptide-nucleic acid unit (PNA) to target the NTD(44–180), flanked by two peptidic regions rich in negatively charged residues to enhance the binding by engaging also the flanking disordered regions, IDR1(1–43) and IDR2(181–248)^20^.

In this scenario our goal was to focus on peptides as possible ligands of this region of the N protein, and to explore different features of the peptides starting from a previously designed one^20^, which was shown to interact with NTD(44–180). Peptides represent ideal candidates for targeting this complex construct due to their design versatility and synthetic tractability. Various features are here investigated including the presence of a large fraction of negatively charged residues, the role of a central aromatic residue, the role of bulky leucine residues as well as the possible distribution of different elements within the primary sequence. We further took advantage of the possibility to introduce in the synthetic peptide a ^19^F nuclear spin in order to have clean information on the peptide-protein interaction in a straightforward way through NMR spectroscopy^26^. After confirming that the design of the starting peptide was suitable to also engage the flanking IDRs in the interaction, we focused on modulating the central part of the peptide to enhance the affinity for the central interacting region of NTD(44–180).

NMR-based analysis, including ^19^F detection, combined with molecular dynamics simulations and circular dichroism, allowed us to explore how these ligands engage the structurally heterogeneous regions of the NTD(44–180). This strategy had the aim of contributing to a deeper understanding of the interaction mechanisms of the N protein and supports the development of fluorinated peptides as molecular probes, opening new avenues for the design of antiviral agents.

## Results

### Do flexible regions play a role in the interaction?

**Fig. 1:**
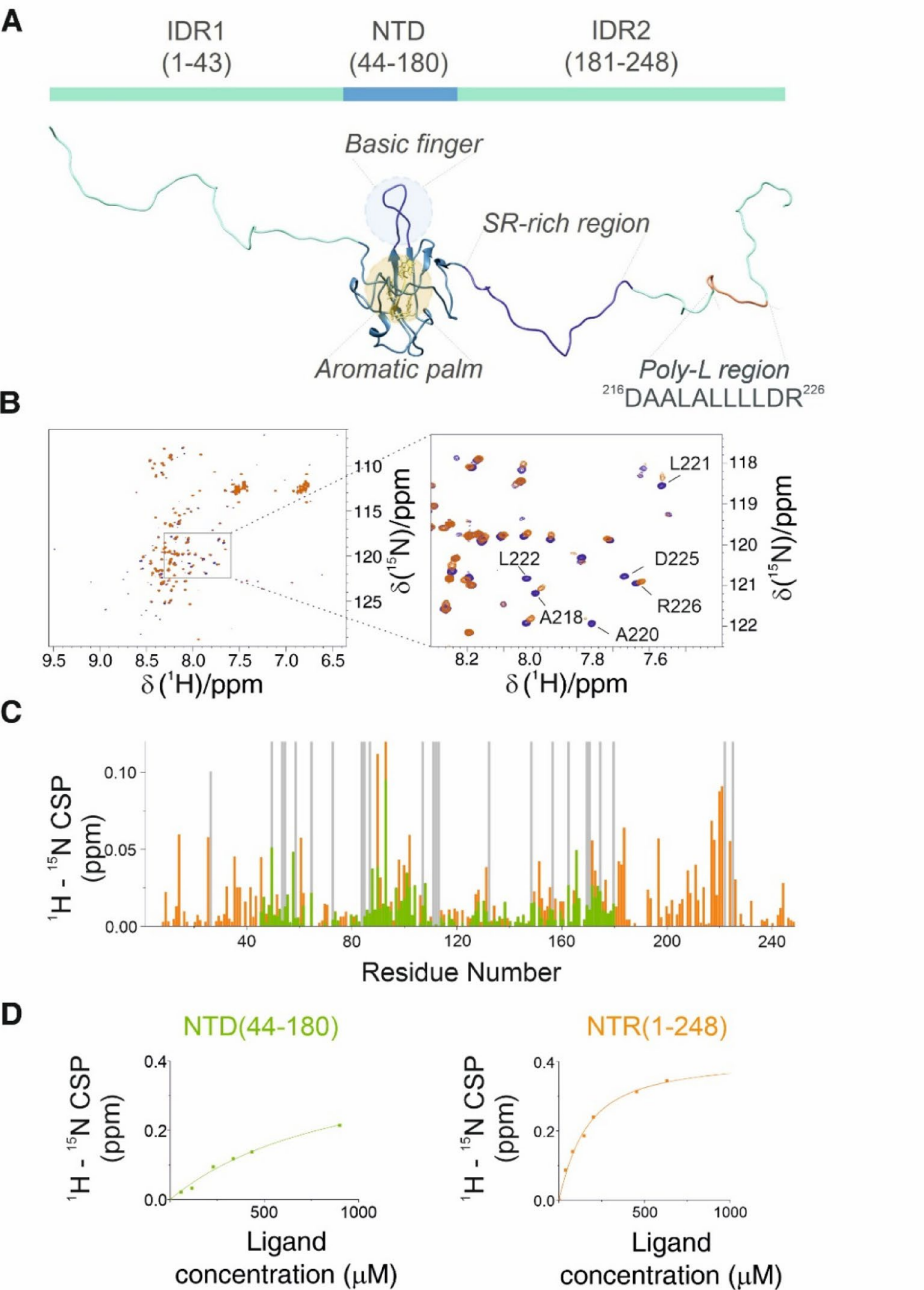
(**A**) Schematic representation of the SARS-CoV-2 N-terminal region (NTR, residues 1–248), showing the organization of its structured and disordered elements: IDR1 (1–43), the folded N-terminal domain (NTD, 44–180), and IDR2 (181–248). The structural model highlights the crucial regions such as the basic finger and aromatic palm (within NTD(44–180)), and SR-rich region (177–203), the poly-leucine tract (residues 216–225) within IDR2; (**B**) Region of the 2D ^1^H-^15^N HSQC NMR spectra of the NTR(1–248) construct showing significant perturbations upon titration with peptide P0. Clear chemical shift perturbations (CSPs) and disappearance of selected cross-peaks are observed. (**C**) CSP plot (orange) highlighting the most affected residues upon addition of peptide P0 (1:1 peptide:protein molar ratio). Grey bars indicate residues for which signal disappearance occurred. The superimposed CSP plot (green) shows for comparison the data obtained for the interaction of P0 with NTD(44–180)^20^. (**D**) Fitting of CSP data obtained from both titrations (NTD(44–180) in green and NTR(1–248) in orange) to estimate the corresponding dissociation constants (*K*_*d*_) for residue 94. The conformer displayed in panel (**A**) has been generated by manually merging one of the conformers of NTD(44–180) present in the 9QWI^27^ PDB structure with two disordered regions, IDR1(1–43) and IDR2(181–248) generated through Flexible Meccano^28^.

To develop a peptide-based binder targeting the NTR(1–248) construct, we evaluated first the main factors driving its interaction with viral RNA^7,8,10,12,14^. As Fig. 1 shows, the NTR(1–248) construct is rich in positively charged residues playing a role in RNA interaction; the heart of this construct is NTD(44–180), a globular region primarily responsible for binding viral RNA. It presents a surface enriched in positively charged residues, especially within the flexible loop comprising residues 90–106, the so called “basic finger”, and its junction with the protein cleft, known as “palm”, which is rich in hydrophobic and aromatic residues particularly in the β3-strand encompassing residues 108–112. The two intrinsically disordered regions flanking the NTD(44–180), i.e. IDR1(1–43) and IDR2(181–248), are also highly flexible and rich in positively charged amino acids and these usually contribute to enhancing the interaction between the protein and RNA and also other tested ligands^19–21^; the 180–206 region, also referred as “SR-rich region” due to the redundancy of serine and arginine residues, is demonstrated to be particularly relevant for the binding^9^.

Based on these observations, the key elements underlying the design of the initial peptide (P0) were: (i) a polyanionic character through multiple glutamate residues to exploit electrostatic attraction; (ii) a central hydrophobic cluster formed by closely spaced leucine residues to interact with the aromatic palm; and (iii) flexible glycine-rich termini to adapt to the modular architecture of the protein, potentially engaging the flanking disordered regions. This resulted in the P0 sequence (Ac-Glu-Gly-Glu-Gly-Glu-Gly-Gly-Leu-Leu-Glu-Leu-Ala-Leu-Glu-Leu-Leu-Gly-Gly-Glu-Gly-Gly-(β-Ala)-Glu-NH_2_).

We therefore studied the interaction of this peptide with the NTR(1–248) construct to evaluate the contribution of the IDRs, and to compare it with the previously reported interaction of the same peptide with the isolated NTD(44–180) domain^20^.

The ^1^H-^15^N HSQC NMR spectrum recorded on NTR(1–248) clearly highlights resonances corresponding to two regions strongly affected upon binding: the SR-rich region (177–203)^9,22^ and the poly-leucine region (216–225) (Fig. 1B). Interestingly the chemical shift perturbations (CSP) observed for ^1^H-^15^N cross peaks in the two intrinsically disordered regions, particularly in the poly-leucine tract, are of the same order of magnitude as those observed for the globular domain, indicating a pronounced role played by the intrinsically disordered regions in this case. Cross peaks deriving from residues in the globular domain also show significant broadening, as a result of increased exchange contributions (inter and/or intramolecular) as well as possible slower tumbling of the complex. A similar behavior was previously observed for NTR(1–248) with different types of polyanions^19–21^.

It is also interesting to compare the results obtained by gradual addition of the P0 ligand to the NTR(1–248) construct, which also includes the flanking IDRs, with the previously determined data for the isolated NTD(44–180) (reported in green in Fig. 1C)^20^. The regions perturbed within the NTD(44–180) are the same also when the IDRs are present, indicating that the same region of NTD(44–180) is perturbed in the two cases. The plots of CSP values versus increasing ligand concentration (Fig. 1D) also highlight an increase in the binding affinity when the IDRs are present as shown through the example of residue 94, a central residue in the NTD(44–180) “flexible finger” involved in the interaction. I94 is one of the most perturbed residues in both titrations and shows a pronounced decrease in *K*_*d*_ in presence of IDRs, when passing from the NTD(44–180) to the NTR(1–248) constructs (NTD(44–180) shows a *K*_*d*_ of 847 ± 222 μM while NTR(1–248) shows a lower *K*_*d*_ of 120 ± 14 μM). Similar results are observed for neighboring residues as shown in the Supplementary Material (Figure S1). The increase in the overall affinity for P0 is thus mediated by the presence of additional flexible modules in the protein.

Summarizing, these novel data on the P0-NTR(1–248) interaction confirm that the design of the peptide is appropriate to engage also the flanking disordered regions of NTR(1–248) in the interaction. The following steps focused on investigating specific features that modulate the interaction with the core of the interacting region, that present on the NTD(44–180) as discussed hereafter.

### Zooming into the central globular domain

We turned our attention to enhancing the interaction of the peptide P0 with the central NTD(44–180) domain, the core region responsible for the native binding to viral RNA, by introducing additional features to enhance the strength of the interaction. In particular, we aimed to introduce a mild π–π stacking capability to mimic the nucleobase–aromatic interactions that are often necessary to RNA–protein binding. To this end, we replaced the central alanine residue in P0 with a para-fluorinated phenylalanine Phe(4-^19^F), thereby introducing both an aromatic moiety and a fluorine atom.

This substitution was expected to enhance affinity towards the aromatic-rich “palm” region of the NTD(44–180) via stacking interactions and to provide a ^19^F NMR-active probe for direct ligand-based binding detection^29–31^. The resulting modified peptide analog, P1, maintained the modular architecture of the original designed P0: Glu and Gly rich flexible termini flanking a more bulky Leu-rich core, now reinforced by an aromatic centerpiece.

To assess the role of primary sequence and modularity, we designed a second peptide P2, with the same amino acid composition as P1 but with a redistributed primary sequence. The central Phe(4-^19^F) residue was retained in the same position to preserve the stacking capability, while Glu, Leu, and Gly residues were homogeneously dispersed along the chain. This allowed us to disentangle the contributions of primary sequence arrangement, secondary structure propensity, and residue clustering from the intrinsic physicochemical properties of the amino acids themselves.

The study of P1 and P2 aimed at evaluating how aromaticity and fluorination contribute to the peptide interaction strength and dynamics, as observed both from the protein and ligand perspectives. The parallel study of the two peptides was also instrumental to understand the optimal conformational properties of the peptides still keeping the same amino acid composition. The designed primary sequences are.

- **P1:** (Ac-Glu-Gly-Glu-Gly-Glu-Gly-Gly-Leu-Leu-Glu-Leu-Phe(4-^19^F)-Leu-Glu-Leu-Leu-Gly-Gly-Glu-Gly-Gly-(β-Ala)-Glu-NH_2_).
- **P2:** (Ac-Gly-Glu-Leu-Glu-Gly-Leu-Glu-Gly-Leu-Glu-Gly-Phe(4-^19^F)-Glu-Leu-Gly-Leu-Glu-Gly-Leu-Glu-(β-Ala)-Glu-Gly-NH_2_).

P1 has been designed to provide a more bulky central region, leaving the edges more flexible (Figure S2A). P2 is instead more homogeneous in the overall distribution of amino acids (Figure S2B). The different amino acid distribution leads to different chemical and structural properties as also predicted by the FELLS software (Figure S2)^32^.

### P1 and P2 conformational sampling

We investigated the conformational landscape of the two designed peptides P1 and P2 using molecular dynamics (MD) simulations. To assess their behavior at both the energetic and atomic levels, we analyzed their free energy landscapes (FEL). However, peptides often adopt locally stable conformations separated by high energy barriers, which limits the ability of conventional MD to capture folding events. Replica exchange molecular dynamics (REMD) addresses this limitation by simulating multiple replicas of the system at different temperatures in parallel. This temperature-driven random walk allows the system to escape from local minima by sampling higher-energy states.

**Fig. 2:**
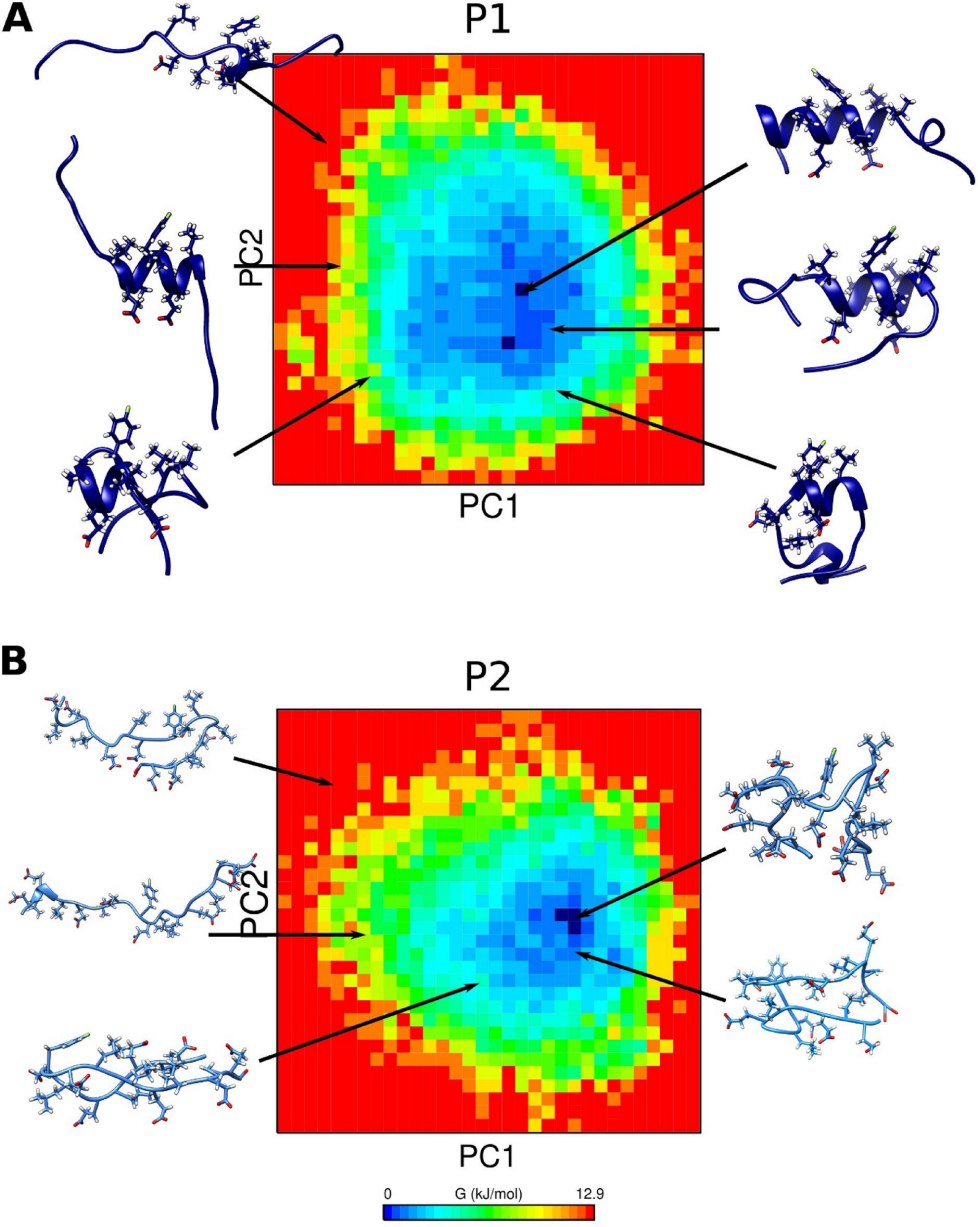
Free energy landscapes and representative conformations of P1 and P2. (**A**) P1 and (**B**) P2 FELs projected onto principal components PC1 (x-axis) and PC2 (y-axis), with energy basins color-coded from red (highest energy) to dark blue (lowest energy). Representative structures are overlaid at key basins, illustrating dominant conformational states.

P1 and P2 FEL contour maps are illustrated in Fig. 2. Principal component analysis was applied to capture the major collective motions in the simulations. Trajectories from the reference replica (298 K) of both REMD runs were projected onto two-dimensional spaces defined by the principal components with the largest eigenvalues. These projections were converted into free energy landscapes, where the most populated regions, corresponding to the lowest free energy, represent the more stable states. The FEL projection presents a broad basin for both peptides. P1 conformation in the lowest energy state is helical, mainly in the N-terminal region, and coiled at the C-terminal one. As shown in Fig. 2A, P1 samples helical conformations in the central region (Leu13-Leu16); residues 1–4 and 17–23 remain flexible and disordered (Fig. 2A). Instead, P2 adopts a coiled conformation in low-energy regions of the free-energy landscape (Fig. 2B). The sampled conformations involve both extended as well as compact states, probably due to the hydrophobic effect driven by the presence of six leucine residues and one phenylalanine in the sequence.

P1 and P2 free energy landscapes reveal distinct differences in their conformational preferences and dynamics (Supplementary Material Figure S3). Notably, P1 exhibits a broader low-energy region (dark blue), indicative of a more stable state. In contrast, P2 displays a more prominent intermediate-energy zone (turquoise), suggesting a shift toward less compact or more dynamic conformations. This distinction aligns with their respective radius of gyration (Rg) profiles, where P1 adopts a compact fold in its most populated basin but spans a wide range of PC1 values, probably due to the flexible C-terminal region (P1 overall Rg value: 15.18 Å vs. P1 Glu5-Leu15 Rg value: 4.19 Å). P2 spans a range of conformations including more compact ones with lower Rg values. Together, these findings highlight how subtle sequence differences between P1 and P2 manifest in different properties.

### Conformational and experimental analysis of P1 and P2

After the design phase, the peptides were synthesized thanks to the reliability of automated solid-phase peptide synthesis^33,34^. Care was taken during the manual coupling of the fluorinated amino acid to ensure its correct insertion. The peptides were then purified to yield high purity, allowing for their subsequent analysis (Supplementary Material Figures S4 and S5).

We carried out their NMR characterization by combining homonuclear (^1^H–^1^H TOCSY and ^1^H–^1^H NOESY) and heteronuclear (^1^H–^15^N HSQC and ^1^H–^13^C HSQC) 2D NMR experiments. We achieved full sequence-specific resonance assignments for both P1 and P2. A few examples of the quality of the data and assignments are reported in the Supplementary Material (Figure S6–S9 and Tables S1 and S2). The NOESY spectra of P1 and P2 do not show intense cross peaks from long range correlations, demonstrating that both peptides are not characterized by a well-defined 3D structure. The NOESY spectrum of P1 provides more intense cross peaks arising from the residues found in the central part of the primary sequence. On the contrary, P2 shows overall less intense cross peaks due to the more homogeneous amino acid distribution*.* Moreover, the spectrum of P1 also shows two sets of negative peaks arising from Glu1 and Glu23, the most flexible residues in the primary sequence. The same effect cannot be observed in the case of P2. This difference is due to the higher compaction of P1 in the central region and to the higher flexibility of this peptide at the edges of the primary sequence. The more homogeneous amino acid distribution of P2 reduces this effect.

**Fig. 3:**
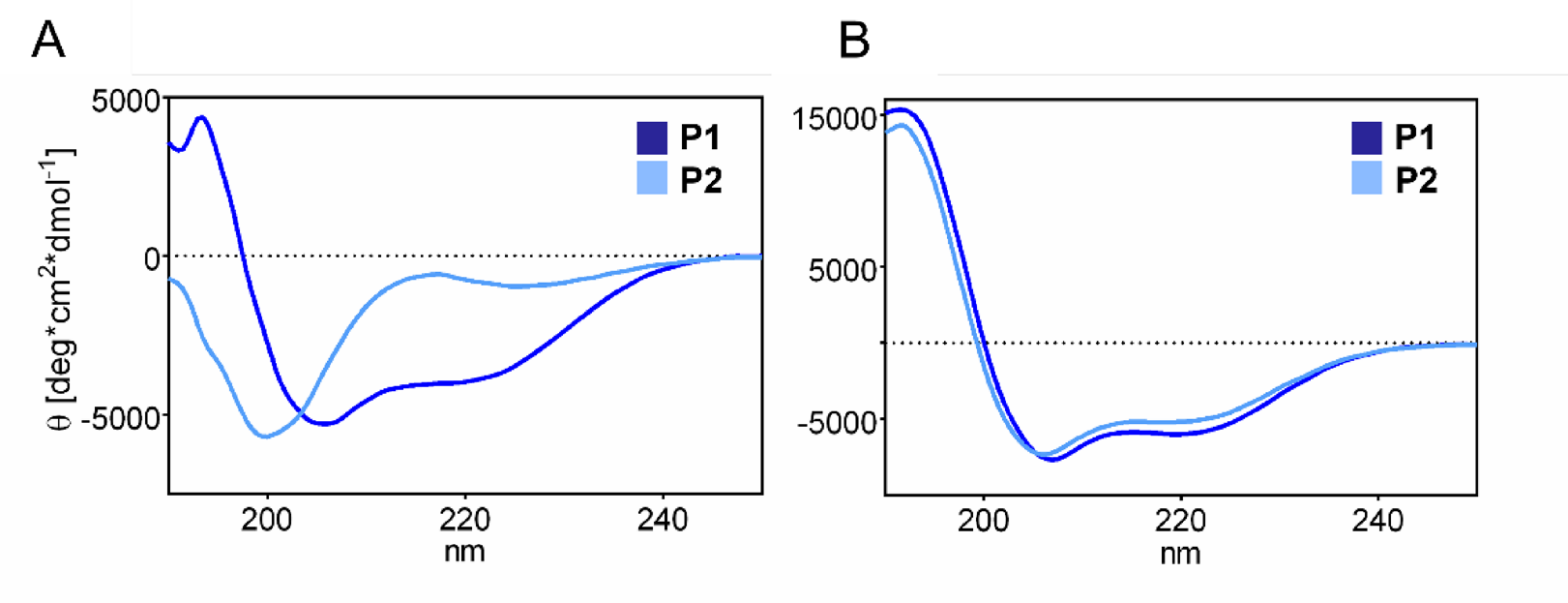
CD spectra of the synthetic peptides, P1 in blue and P2 in light blue, respectively. (**A**) peptides in water solution; (**B**) peptides in TFE:water mixture (50:50). Each curve is the sum of 5 scans. All data were collected at 298 K.

Circular dichroism (CD) spectroscopy was integrated to investigate the secondary structural features of the designed peptides, both in aqueous and in 50% trifluoroethanol (TFE) solutions. As shown in Fig. 3A, P1 displays pronounced minima around 208 and 222 nm, hallmarks of an α-helical propensity even in a highly hydrophilic environment, where peptides typically exhibit increased conformational flexibility. In contrast, P2 does not show a comparable effect, confirming that the arrangement of Leu residues plays a critical role in influencing the secondary structure propensity of the ligands. The fraction of helical versus random coil structure can be estimated by CD using the approach reported in literature^35^. In this case, P1 has values of 19% and 26% in H_2_O and 50% TFE, respectively (Fig. 3A), while P2 has a value of 24% only in 50% TFE (Fig. 3B).

Taken together these data confirm that the two peptides are characterized by different structural and dynamic properties in line with predictions obtained through molecular dynamic calculations.

### Dual-perspective analysis of peptide-protein interactions enabled by ^19^F-ligand monitoring

**Fig. 4:**
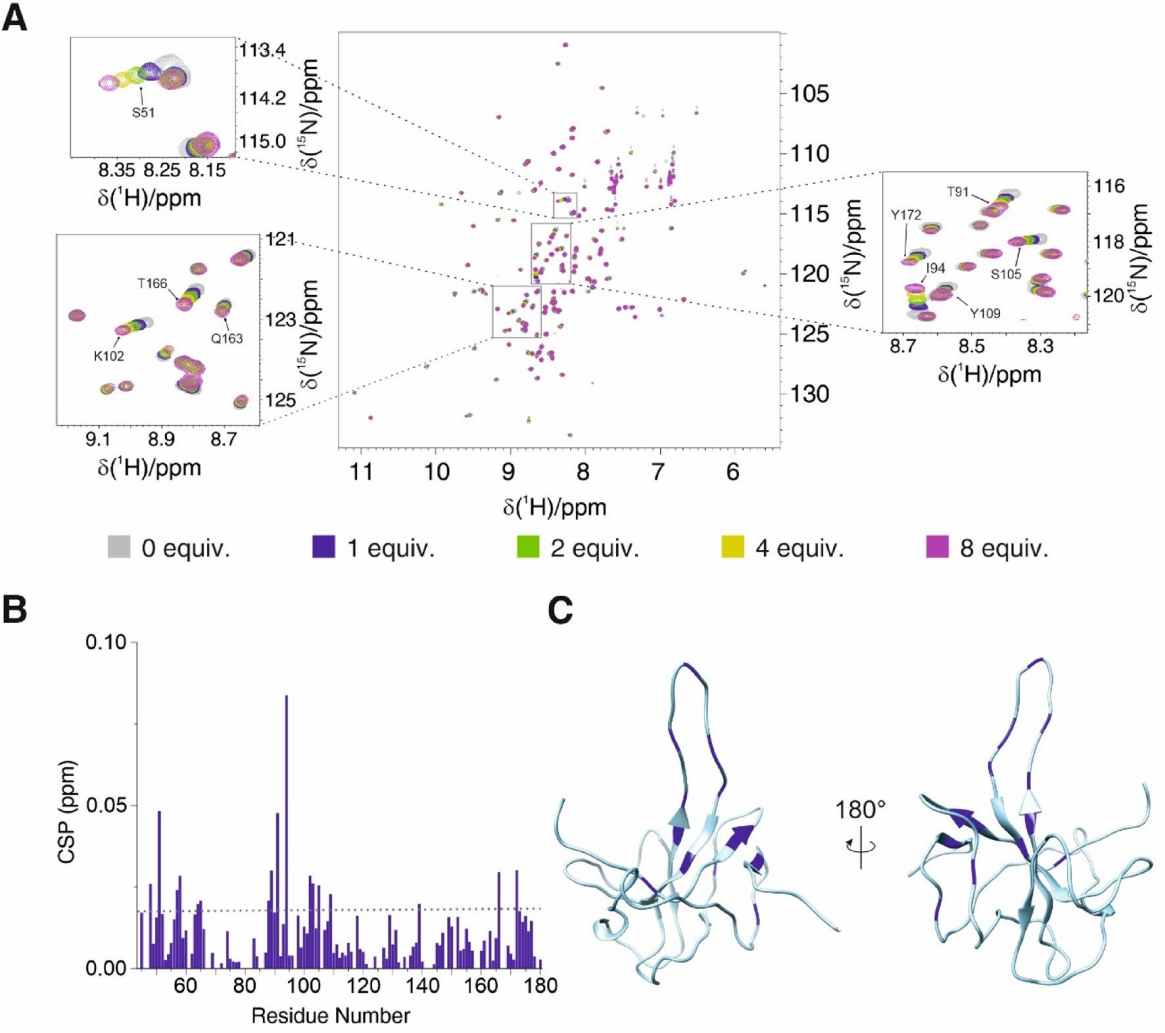
NMR titration of NTD(44–180) with P1. Panel (**A**) displays the superimposition of the reference ^1^H-^15^N HSQC spectrum (in gray) of NTD(44–180) with spectra recorded after addition of P1 in molar ratios of 1.0, 2.0, 4.0, and 8.0 respect to NTD(44–180) (shown in blue, green, yellow, and magenta, respectively), along with zoomed-in view of particularly perturbed regions. Panel (**B**) reports the CSP occurring upon the addition of P1 in 1:1 molar ratio respect to NTD(44–180), highlighting three main affected regions of the protein construct. The most perturbed residues are defined as those whose CSP values exceed the threshold of the mean plus one standard deviation (grey dotted line). These residues (S51, T57, Q58, K65, R89, T91, I94, D98, K102, D103, S105, Y109, T166, Y172) are visualized in blue on the protein frame (PDB 6YI3)^8^ in panel (**C**).

To monitor the interaction between the NTD(44–180) construct and the two peptides P1 and P2, solution NMR titrations were performed. The observation of the changes in cross peaks position in 2D ^1^H-^15^N HSQC spectra allowed us to identify the most perturbed regions in the protein construct. Adding P1 to a sample of NTD(44–180) in a 1:1 peptide:protein molar ratio (Fig. 4A), we observed shifts in the protein cross peak positions, indicating an interaction occurring in the fast exchange regime on the NMR time scale. Analysis of the ^1^H and ^15^N chemical shifts revealed that the most affected residues by the interaction are predominantly clustered in three main regions (51–65, 90–111, 166–173), as reported in Fig. 4B and C. These regions closely resemble those identified upon interactions with RNA^10,12^.

**Fig. 5:**
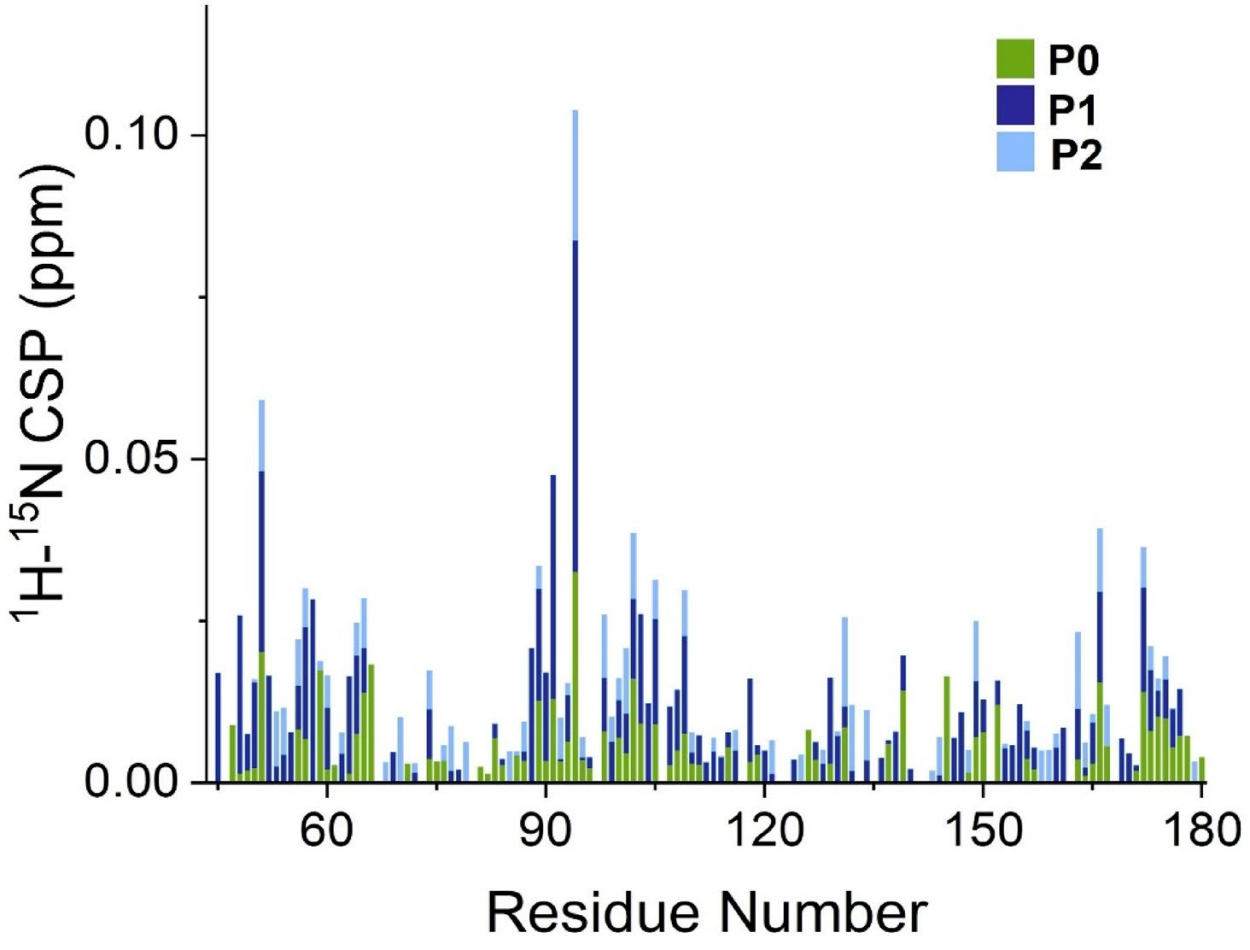
Superimposition of the CSP plots corresponding to 1:1 peptide:protein molar ratio for P1 in blue, P2 in light blue and P0 in green. The introduction of the fluorinated phenylalanine in place of an alanine contributes to an increase of the CSP values, as observed comparing the data obtained for P0 (green)^20^ with those obtained for P1 (blue) and P2 (light blue).

An analogous titration and data analysis was conducted for P2; the protein regions affected by the interaction are nearly the same as for P1 (Fig. 5). For comparison the figure displays also the CSP plot for P0 which shows much smaller changes in chemical shifts (the average calculated CSP for the most perturbed common residues is 0.051 ± 0.007 for P2 and only 0.017 ± 0.007 for P0). These results clearly confirm the importance of introducing an aromatic residue in the central part of the peptide to increase binding affinity. To further confirm this trend we have calculated the *K*_*d*_ values for the most perturbed residues in the three cases (Table S3). The results show that P2 has a higher affinity for the protein with respect to P1 (Figure S10) and that both of them have significantly higher affinity with respect to P0.

Interestingly, residues in the same sequence position in titrations with different peptides show a trend: the binding affinity increases moving from P0 to P1 to P2; of note, different residues in the same titration experiment provide similar but not identical *K*_*d*_ values. There is no clear relationship between the *K*_*d*_ and the residue type.

To complement the protein-centered view provided by CSP analysis obtained through ^1^H-^15^N HSQC experiments, we then monitored the interaction from the ligand perspective by exploiting the sensitivity of ^19^F NMR.

The incorporation of a fluorine atom within the ligand indeed allowed to monitor the interaction also from the “other side” in a straightforward and efficient way. To this end ^19^F chemical shifts and ^19^F transverse and longitudinal relaxation rates were measured while performing the titration experiments^36–38^.

**Fig. 6:**
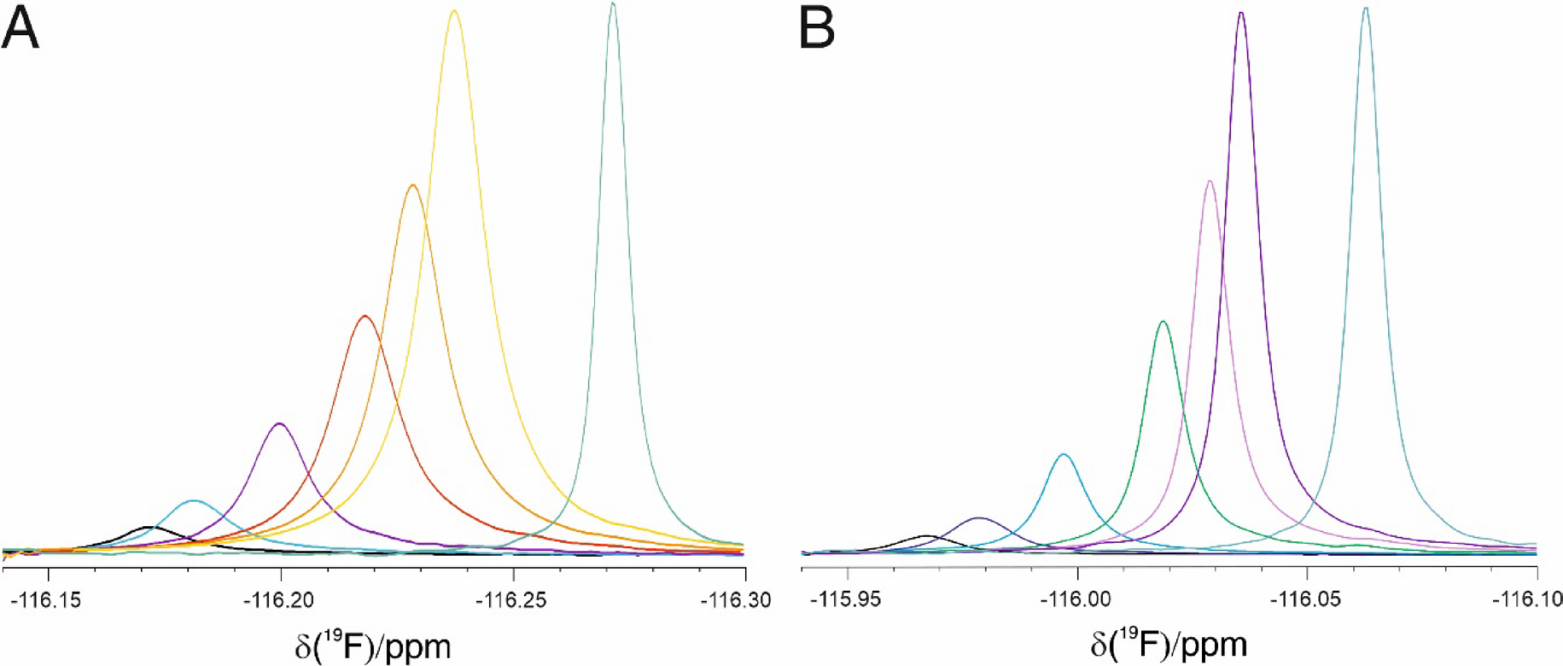
Panel (**A**) shows the superimposition of ^19^F 1D spectra of P1 in the presence of NTD(44–180) in the following NTD(44–180):peptide molar ratios: 1:0.5 (black), 1:1 (cyan), 1:2 (purple), 1:4 (red), 1:6 (orange), 1:8 (yellow). Panel (**B**) shows the superimposition of ^19^F 1D spectra of P2 in the presence of NTD(44–180) in the following NTD(44–180):peptide molar ratios: 1:0.5 (black), 1:1 (blue), 1:2 (cyan), 1:4 (green), 1:6 (pink), 1:8 (purple). In each panel the spectra of P1 and P2 are also reported for reference (on the right of each panel).

The 1D ^19^F NMR spectra of the peptides present a single resonance deriving from the ^19^F nucleus located at position 4 of the benzyl side-chain of Phe12 within both P1 (− 116.26 ppm) and P2 (− 116.09 ppm). The slight chemical shift difference observed for the two peptide signals is due to differences in their chemical environment. We titrated NTD(44–180) by gradually adding increasing amounts of each peptide and monitored the changes of the ^19^F signal upon peptide addition. Figure 6A presents a superimposition of 1D ^19^F spectra recorded for P1 at different protein-to-peptide molar ratios, obtained by progressively adding P1, while maintaining a constant protein concentration.

**Fig. 7:**
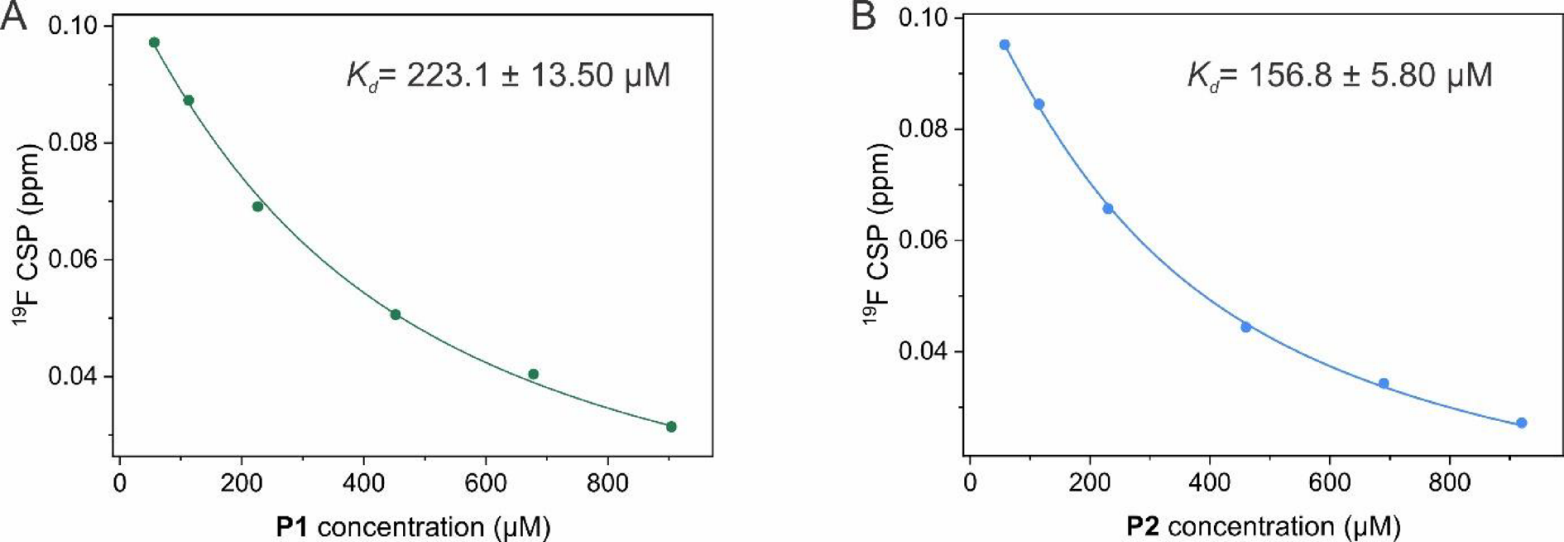
Panel (**A**) reports the chemical shift of the ^19^F as a function of the concentration of P1 and displays the *K*_*d*_ fitting together with its calculated value (223.1 ± 13.50 μM). Panel (**B**) reports the chemical shift of the ^19^F as a function of the concentration of P2 and displays the *K*_*d*_ fitting together with its calculated value (156.8 ± 5.80 μM).

As illustrated in Fig. 6A, when NTD(44–180) and P1 are present in a 1:0.5 molar ratio respectively, the highest CSP is observed with respect to the reference spectrum of P1 alone. The addition of increasing amounts of the peptide to the NTD(44–180) protein construct leads to a progressive decrease in CSP values. This phenomenon can be attributed to an excess of peptide, which shifts the thermodynamic equilibrium towards the free form. A similar analysis was performed also for P2. NMR 1D ^19^F spectra of P2 throughout the titration are shown in Fig. 6B. The obtained *K*_*d*_ values are 223.1 ± 13.50 μM for P1 (Fig. 7A) and *K*_*d*_ = 156.8 ± 5.80 μM for P2 (Fig. 7B).

These results indicate that P2 binds more tightly to NTD(44–180) than P1, in agreement with the trends observed from the analysis of protein backbone ^1^H–^15^N CSPs (Table S3).

^19^F NMR relaxation measurements were also performed to further characterize the interaction of the two peptides (P1 and P2) with the NTD(44–180). Longitudinal relaxation was measured through an inversion recovery approach while transverse relaxation was determined through CPMG NMR experiments. Experimental details are provided in the Supplementary Material and in Figure S11.

For each peptide, *R*_2_ and *R*_1_ values were measured for three different peptide-protein concentration ratios. In the case of P1, we measured an *R*_2_ of 5.26 ± 0.24 s⁻^1^ in the free form, 25.3 ± 0.76 s⁻^1^ in the presence of NTD(44–180) at a 1:1 ratio, and 19.4 ± 0.47 s⁻^1^ at a 1:8 molar ratio (NTD(44–180):P1, Table S4). For P2, the *R*_2_ values were 5.27 ± 0.07 s⁻^1^ in the free state, 15.2 ± 0.27 s⁻^1^ for the 1:1 molar ratio, and 9.73 ± 0.14 s⁻^1^ for the 1:8 molar ratio (Table S5). *R*_1_ values instead are very similar in the three cases (Supplementary Material, Figure S12–S14, Tables S6–S7).

**Fig. 8:**
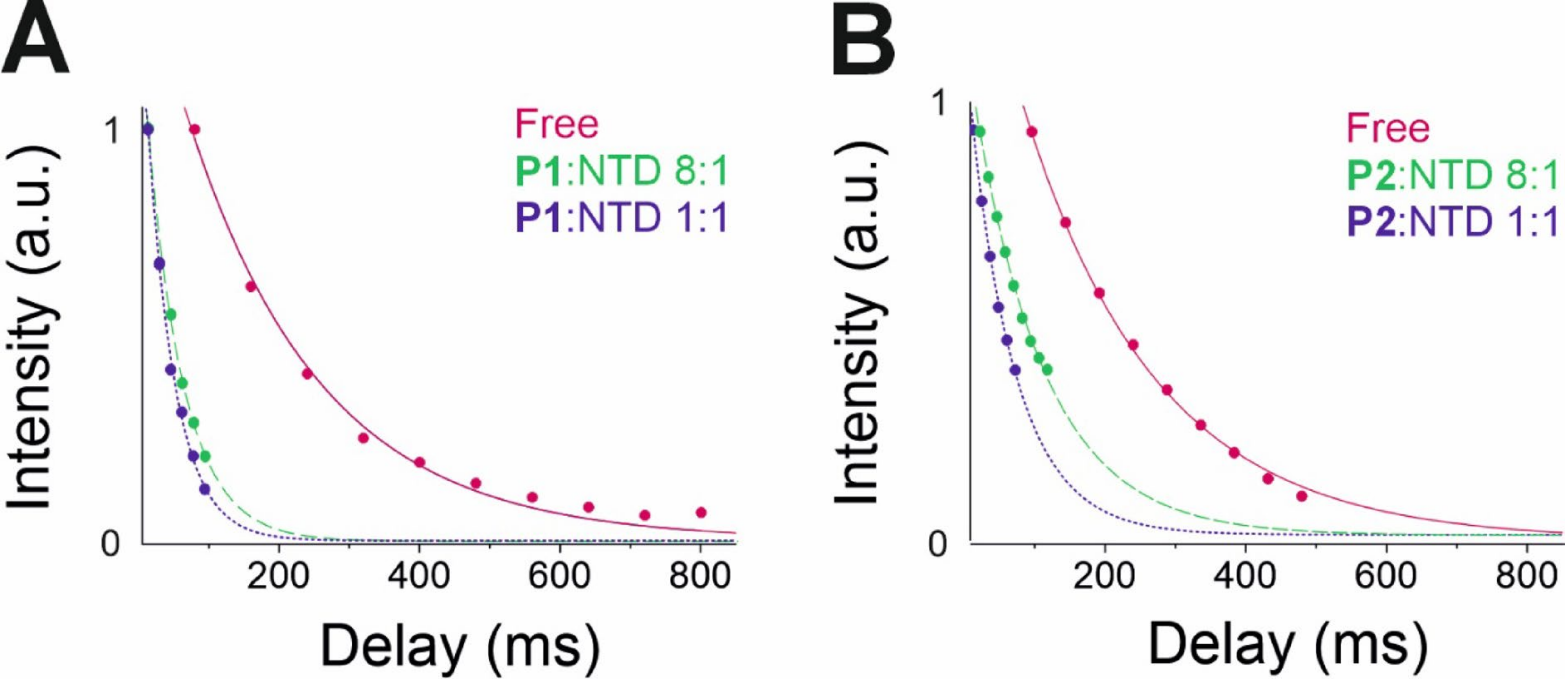
The figure shows on panels **A** and **B** the ^19^F CPMG decays of P1 and P2 respectively. Free form (red), NTD(44–180) and peptides in 1:8 molar ratio (green), and NTD(44–180) and peptides in 1:1 molar ratio (blue) are reported as intensity ratios with respect to the first point of each series of measurements (vertical axis from 0 to 1).

As expected, upon binding, the peptide forms a complex with a larger molecule (i.e., the protein), characterized by a larger rotational correlation time (τ_c_). This results in a significant increase in the effective correlation time experienced by the peptide when bound to the protein, leading to a marked increase in *R*_2_; an additional contribution to the increase of *R*_2_ may also come from exchange processes (either inter or intra molecular ones) sensed by the peptide between the free and bound state. Moving from a 1:1 (NTD(44–180)-peptide) molar ratio to a 1:8 molar ratio leads to a shift of the curve towards that of the free peptide, consistent with an increased population of the unbound form. Taken together these data confirm the interaction of the two peptides with NTD(44–180) and show an increased exchange contribution for P1 respect to P2 (Fig. 8).

### Peptides-protein interaction mode described by MD simulation

**Fig. 9:**
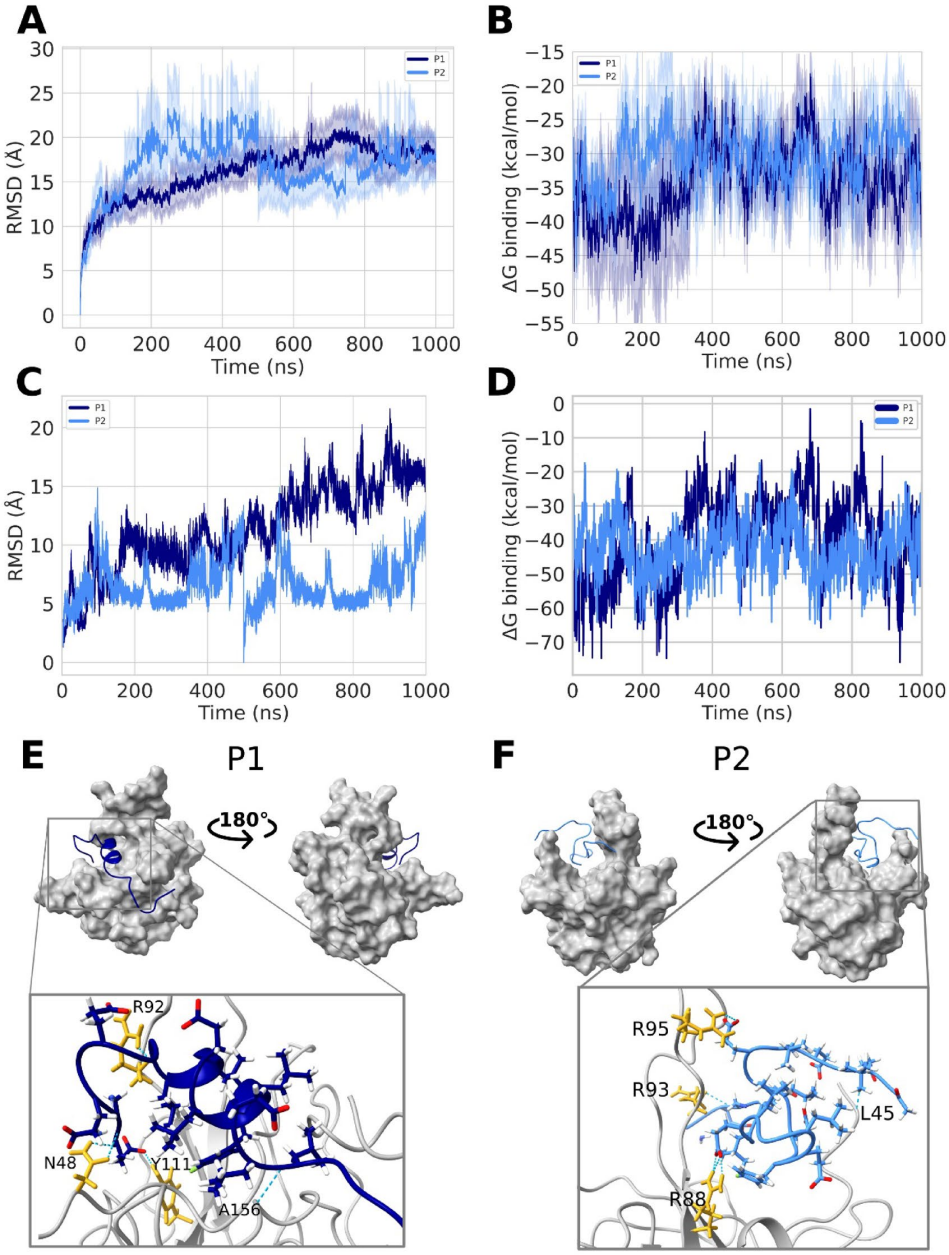
P1 and P2 interaction with SARS-CoV-2 NTD(44–180). (**A**) Average RMSD over 8 independent MD simulations for each peptide (8 for P1 and 8 for P2) of 1 μs. (**B**) Average Generalized Born Electrostatic Energy over the simulations. (**C**–**F**) Analyses based on the MD trajectories initiated from the highest-affinity docking pose for each peptide (one trajectory for P1 and one for P2). (**C**) Peptide-protein interaction RMSD with single references (highest docking binding affinity). (**D**) Average Generalized Born Electrostatic Energy of reference conformations. Panels (**E**) and (**F**) show a 3D structural representation of the complexes of NTD(44–180) with P1 and P2, respectively. On top of each panel a surface visualization of the NTD(44–180) is shown in the complex with P1 (blue ribbon model, panel (**E**) or with P2 (light blue ribbon model, panel **F**). Two views on each complex, rotated by 180° are shown for clarity. On the bottom of each panel a zoom on the key interaction region shows more details by reporting the two peptides (P1 in blue and P2 in light blue) and the NTD(44–180) (the backbone is shown in grey, and the side chains forming hydrogen bonds with the peptides are highlighted in yellow with contacts indicated by dashed lines).

We employed AutoDock Vina^39^ to dock each peptide to NTD(44–180), and used the lowest-energy conformations obtained for REMD as input. The resulting binding poses are shown in Figures S15-S16. To comprehensively evaluate the binding behavior, we conducted eight independent MD simulations of 1 µs each for the two peptides, starting from distinct initial peptide–protein configurations (Fig. 9).

To evaluate peptide deviation from the starting position throughout molecular dynamics simulations, we took the average intermolecular root-mean-square deviation (RMSD) and its standard deviation. These are presented in Fig. 9A. P1 reached a plateau after 700 ns, while P2 reached a plateau after 500 ns. Although P2 starts with greater RMSD values than P1, which indicates that P2 starts with more conformational changes than P1, both peptides converge to similar average RMSD values around 18 Å over the course of the simulations, driven by a gradual increase in P1 RMSD. These values represent averages across 8 independent simulations for each peptide. The convergence suggests that, despite differences in their initial conformations, both peptides stabilize to similar states upon interacting with NTD(44–180). However, this similarity in RMSD does not necessarily reflect equally favorable interactions: it may instead result from a mixture of optimal and suboptimal binding.

Analyzing the simulations, we can observe that both peptides undergo conformational changes to accommodate in the protein binding region when interacting with NTD(44–180). The overall binding energy shows minimal difference, with P1 at − 34.25 ± 15.75 kcal/mol and P2 at − 30.72 ± 17.54 kcal/mol. The per-residue contributions to the binding energy (van der Waals, electrostatic, polar) are reported in Figure S17.

Subsequent analysis focused on the peptide starting pose associated with the highest binding affinity. P2 RMSD values remain around 5 Å for most of the trajectory, while P1 goes from 10 to 15 Å (Fig. 9C). Generalized Born (GB) binding energy calculations showed favorable binding energetics, with P1 (ΔG = − 29.11 ± 7.07 kcal/mol) demonstrating weaker electrostatic interactions compared to P2 (ΔG = − 46.60 ± 7.76 kcal/mol) (Fig. 9D). This suggests that both peptides have favorable binding to NTD(44–180), but P2 has a stronger binding. P2 has slightly stronger charge-charge interactions (− 1165.69 ± 81.95 kcal/mol) than P1 (− 1138.91 ± 105.53 kcal/mol) and better hydrophobic packaging (− 67.8 ± 7.525 vs − 53.0 ± 8.640 kcal/mol). Altogether, these data suggest that P2 forms more charged interactions and hydrophobic contacts than P1. The conformations of the peptides-NTD(44–180) protein complexes were clustered (Table S8) and the representative structure of the most populated cluster is represented in Fig. 9E–F, for P1, and P2, respectively. Hydrogen bonds with an occupancy greater than 20% throughout the MD trajectories were identified using cpptraj and are reported in the Supplementary Material (Figure S18). P2 forms hydrogen bonds with residues located on the NTD(44–180) surface, including Arg88, Thr91, Arg92, Arg93, Arg95, Tyr111 and Asp128, while P1 forms hydrogen bonds with residues Asn48, Trp52, Arg88, Arg92, Tyr109, Tyr111 and Arg149. In summary, arginine residues and Tyr111 are consistently involved in peptide interactions in the MD simulations.

**Fig. 10:**
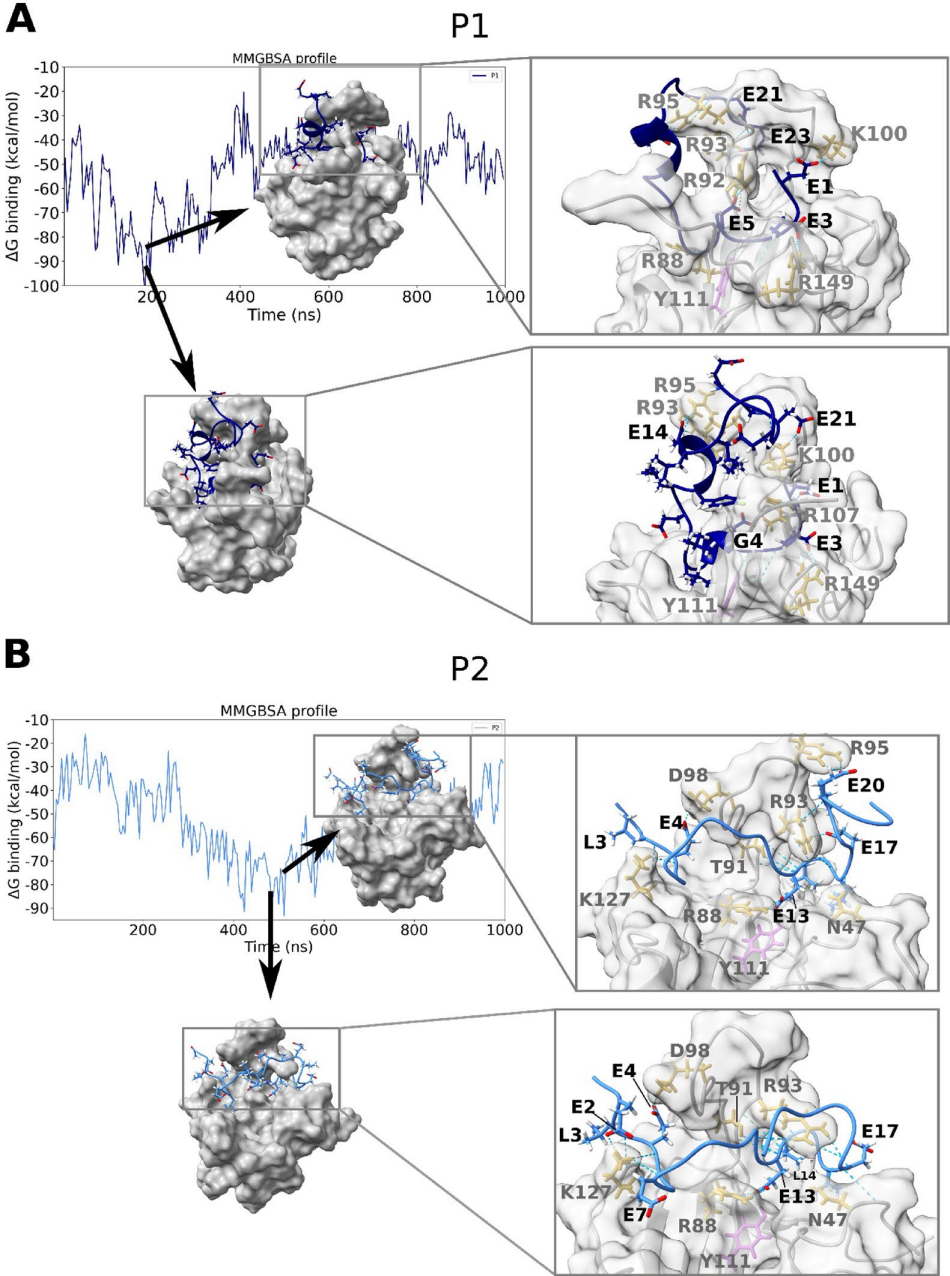
Peptides interaction with NTD(44–180) at the lowest energy time instants. The figure represents the MMGBSA (molecular mechanics generalized Born surface area) profile of the peptides P1 (**A**) and P2 (**B**) during the 1 microsecond trajectory. For each peptide, two conformations are exhibited, highlighting the “pincer grasp” from NTD(44–180) with the peptide inside. The zoom region focuses on side-chains interactions (P1 in dark blue, P2 in light blue). NTD(44–180) protein residue labels are colored in gray, while the peptides residue labels are colored in black. NTD(44–180) side chains are colored in yellow, except for the aromatic residues, colored in purple/orchid. Hydrogen-bonds are represented by dashed-lines in light-blue; For P1 in the first cluster, we can observe hydrogen bonds between the residues in the pairs R92-E5, R95-G20, I94-E21, R93-E23, R93-E21, R95-E14, R95-E21, R92-E5, K100-E1, R149-E3, S51-E3, Y111-G4 and R88-G7. The second cluster also evidences new interactions between the residues in the pairs T49-E5, R93-E14, R107-E1 and K100-E21 (**A**). For P2, we can list: N47-L16, R88-E13, T91-E13, R93-L14, R93-L19, R93-G15, R93-E17, R95-E20, K100-E4, Y111-E13, K127-E4, D128-L3 and L14-T91, for the first cluster, and additional R93-G11, R93-G18, K127-E2, K127-E7 and P46-G18 for the second cluster (**B**) (Table S9).

The evaluation of the peptide binding to NTD(44–180) from 8 different starting points for each peptide, reveals insights into the preferential mode of interaction for P1 and P2. The first observation that emerges inspecting the different trajectories is that electrostatic interactions are the primary driving forces promoting the interaction between the peptides and NTD(44–180), mainly between Glu residues from the peptides and Arg residues from the NTD(44–180) finger part. The tightest interaction (ΔG binding levels at -90 kcal/mol) occurs when the NTD(44–180) basic finger engages the peptide with a partial structural rearrangement to accommodate it, assuming a conformation similar to the hand “pincer grasp” (Fig. 10). In these conditions, the majority of the inter-molecular hydrogen bonds are formed.

A detailed analysis was conducted also on the two most representative clusters for each peptide-NTD(44–180) complex using the hydrogen bond analysis module of AMBER^40^. The complete list of the observed contacts is reported in Table S9. In summary, for P1, the analysis mainly revealed hydrogen bonds between residues with opposite charges (mainly positively charged arginine residues of NTD(44–180) interacting with negatively charged glutamate residues of P1). For P2, a broader distribution of hydrogen bonds was found (Table S9). These hydrogen bonds comprise backbone contacts involving also a few hydrophobic residues.

The presence of peptide-protein salt bridges was also evaluated and several high-occupancy salt bridges (> 50% of the trajectory) at the peptide-NTD(44–180) interface were identified (Table S10). Both P1 and P2 showed salt bridges involving glutamate residues of the peptide interacting with arginine and lysine residues of NTD(44–180). Taken together, these results indicate that the salt bridge and hydrogen-bond network provide electrostatic stabilization for the peptide ligands.

### The role of the aromatic region in the NTD(44–180) protein-peptide interaction

**Fig. 11:**
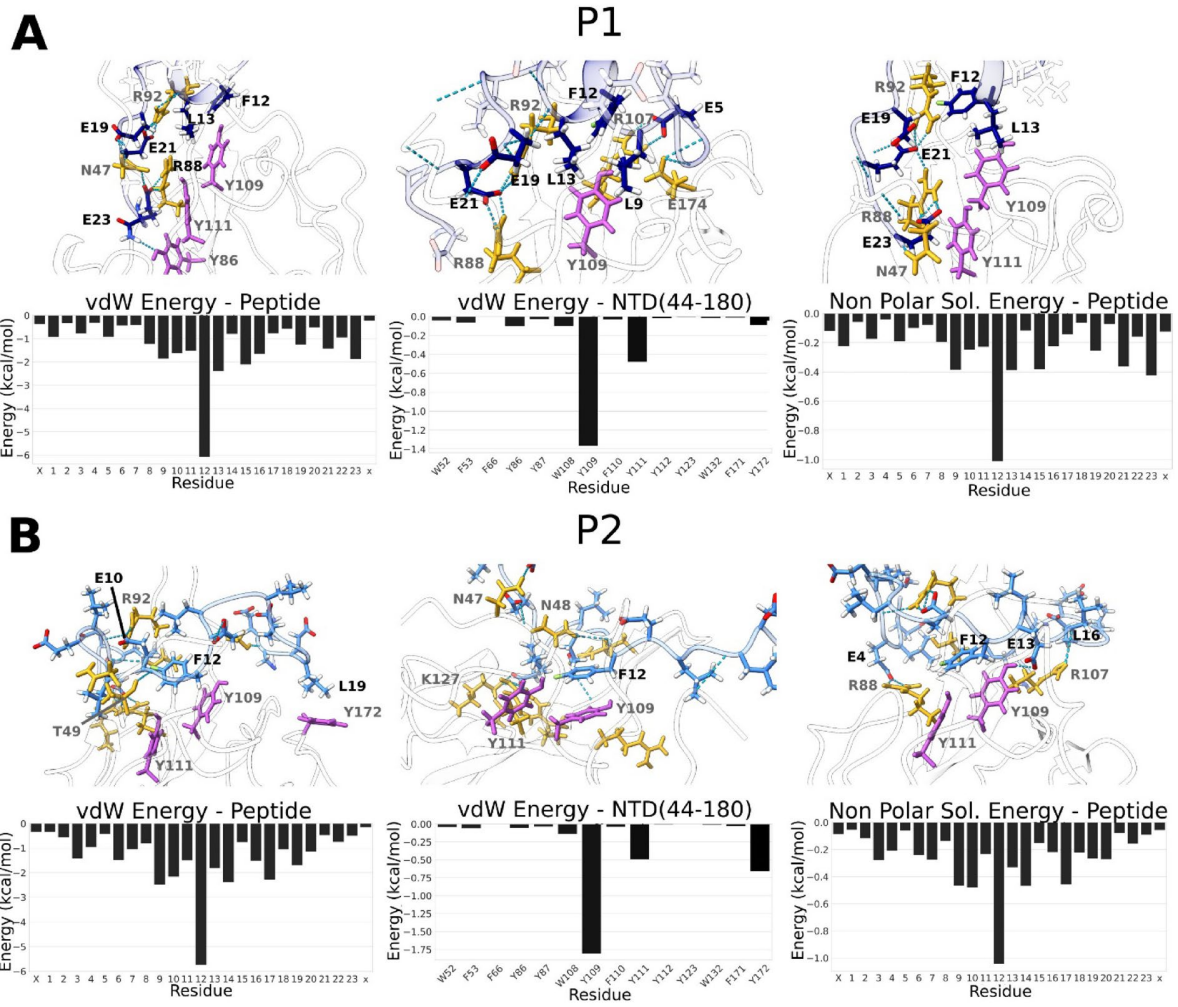
Peptide interaction with aromatic residues of NTD(44–180) protein. Snapshots of peptide-protein interaction and energy plots for van der Waals and non-polar solvation for P1 (**A**) and P2 (**B**) (P1 in dark blue, P2 in light blue). NTD(44–180) protein residue labels are colored in gray, while the peptide residue labels are colored in black. NTD(44–180) side chains are colored in yellow, except for the aromatic residues, colored in purple/orchid. Hydrogen-bonds are represented by dashed-lines in light-blue.

The aromatic residues in NTD(44–180) are mainly present at the base of the NTD(44–180) fold, the NTD(44–180) palm, below the basic finger. To investigate if the peptides also interact with the aromatic residues, we analyzed the contacts (lower than 4 Å) between the peptides and the aromatic residues from NTD(44–180) (Fig. 11). Overall, the main residues interacting with both peptides are Tyr109 and Tyr111 in all trajectories. For P1, we can mention the interaction of Y109 with Phe12 and Leu13, and Tyr111 with Glu23, (Fig. 11A). These connections are stabilized mainly by other peptide connections: Arg88, Asn47, and Arg92 in the NTD(44–180) side, with Glu residues from P1: Glu23, Glu21, and Glu19. Energy plots for van der Waals interactions show the main contribution of Phe12 in the peptide, and Tyr109 and Tyr111 in the NTD(44–180) protein.

For P2, we can also observe Tyr109 and Tyr111 in NTD(44–180) interacting with Phe12. Also, here we observed interactions of Phe12 with Tyr109, indicating the possible formation of π–π stacking interactions (Fig. 11B). Other residues forming hydrogen-bonds with the peptide are: Asn47, Asn48, Arg 88, Arg 92, Arg 107 and Lys127.

In conclusion, it is worth noting that the H^N^-based NMR experiments that we have conducted focus on the backbone of the protein providing evidence of the interaction and highlighting the main binding region(s). However, these data do not necessarily reflect the direct involvement of residue side chains which are in many cases relatively long (e.g. those of arginine, lysine, tyrosine side chains) and can be involved in hydrogen bonding in quite distant parts of the protein with respect to the backbone of such amino acids. In contrast, MD can provide insight into the behavior of the side chains of the residues directly involved in the interaction. Despite this fundamental difference in the approach, the main regions identified as interacting by NMR and by MD simulation show a good agreement for the two peptide-NTD(44–180) complexes.

## Discussion

One of the main functions of N protein consists in packing RNA. The latter is constituted by an extended and negatively charged backbone, a property that can be mimicked by polyanions. The potential of a peptide sequence as a possible ligand of the NTR(1–248) is explored in this work, starting from the previously reported P0 sequence (Ac-Glu-Gly-Gly-Gly-Glu-Gly-Gly-Leu-Leu-Glu-Leu-Ala-Leu-Glu-Leu-Leu-Gly-Gly-Glu-Gly-Gly-(β-Ala)-Glu-NH_2_)^20^.

The comparison between the interactions of P0 with NTD(44–180) and with NTR(1–248) revealed notable differences. While both constructs showed perturbations in similar core binding regions, the titration with NTR(1–248) also highlighted the role of specific regions within the two intrinsically disordered regions flanking the central globular domain. These results demonstrate that the peptide design is appropriate to engage also IDRs in the interaction.

We then focused on improving the original design targeting the central RNA-binding site located in the globular domain (NTD(44–180)). The two new peptide sequences, P1 and P2, were developed in the present study to explore how structural arrangement, aromatic contribution, and conformational flexibility could impact the interaction with the protein. A single para-fluorinated phenylalanine residue was introduced into the central region of both sequences to introduce a minimal aromatic feature present in RNA bases. This approach offered a peptide alternative to our previously developed peptide-PNA chimera^20^, where nucleobase mimicry was achieved directly^41^. In the present study, our aim was to explore whether a simpler, peptide ligand can still provide effective interaction, offering potential advantages in terms of synthesis^42^, and applicability.

In particular, the insertion of the fluorinated phenylalanine enabled fluorine-based NMR detection and represents a key strength and major improvement of our work allowing us to study these interactions from a dual perspective: not only from the target protein point of view, but also from the desired ligand. Moreover, this study presents the characterization of the structural and dynamic behavior of P1 and P2 using an integrative approach that combines molecular dynamics simulations, circular dichroism, and NMR spectroscopy.

All the techniques indicated that both peptides interact with the same target region on the NTD(44–180) with higher affinity respect to P0 confirming the importance of the central ^19^F-Phe residue; yet P2 showed a slightly stronger interaction. Molecular dynamics in particular showed a lower overall ΔG for P2. Interestingly, while P2 exhibited more favorable electrostatic interactions, P1 demonstrated van der Waals contacts and non-polar solvation, highlighting how different structural arrangements can balance binding energy components. Circular dichroism revealed a clear α-helical propensity in P1 even in aqueous solution, likely due to the high density of Leu residues in the middle of the sequence. NMR analyses corroborated these differences and enabled atomic-resolution insight into peptide–protein interactions. We also exploited ^19^F direct detection and performed ^19^F relaxation experiments. Clear differences in the relaxation behavior between the free and bound states of both peptides were observed, confirming complex formation and changes in dynamic properties upon binding. CSP analysis of P2 showed a higher binding affinity, as observed independently from both protein-based and fluorine-based chemical shift perturbations. These results, where P2 binds more tightly, suggest that both the primary sequence and the secondary structure may play a decisive role in stabilizing the complex. The modular nature of P1 despite the more favorable electrostatics and intrinsic helical structure, is likely not necessary for the interaction with NTD(44–180). A more elongated and flexible core, as in P2, appears more potent to fit in the region between the finger and the palm.

**Fig. 12:**
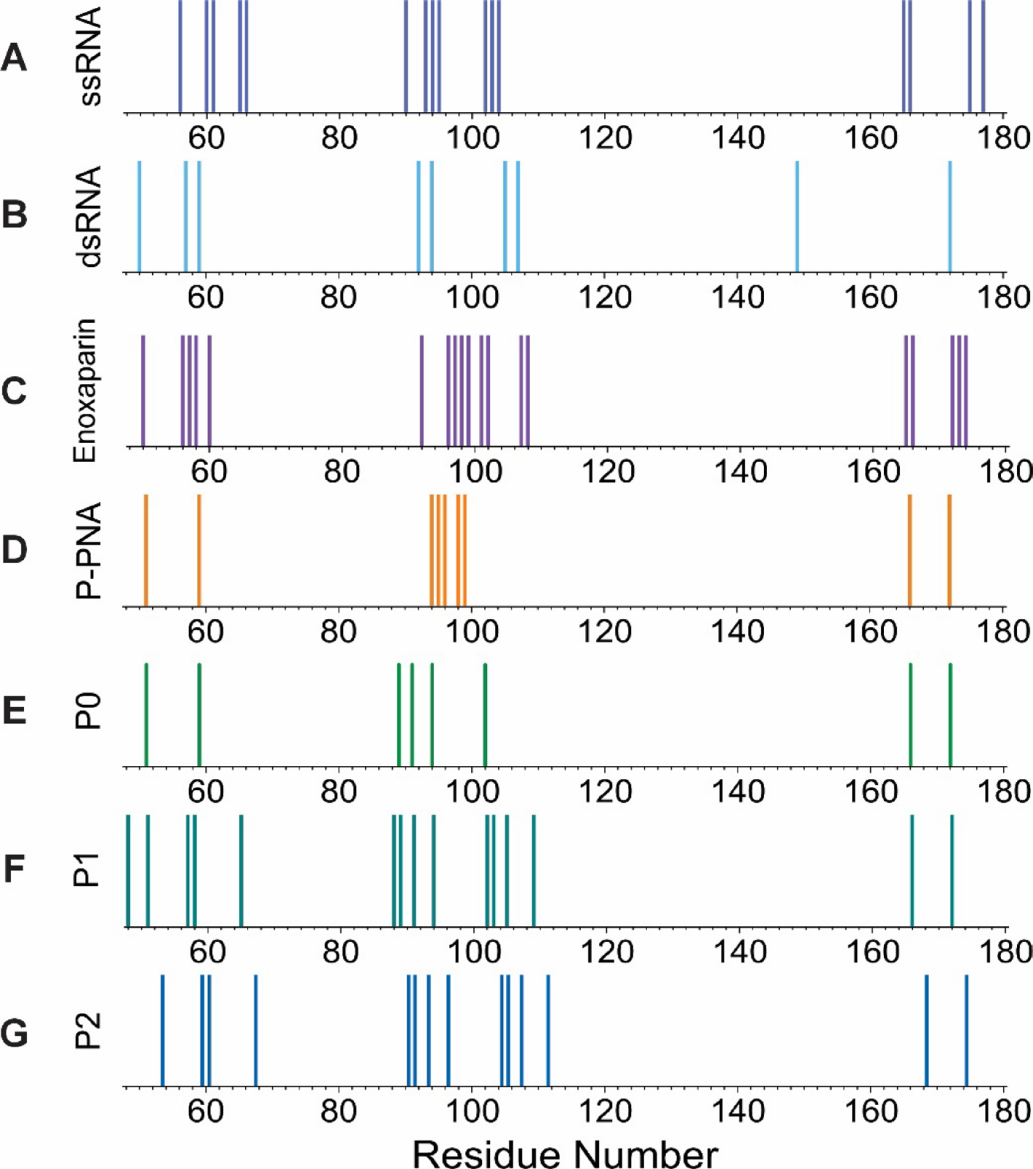
Comparison of CSP profiles obtained upon titration of NTD(44–180) with different ligands; vertical lines indicate residues showing significant perturbations. Each panel corresponds to a different ligand: (**A**) ssRNA: 14-mer single-stranded RNA: 5′-GGCACAUGGACGUC-3′.^12^ (**B**) dsRNA: Double-stranded 14-mer obtained by annealing the ss-14mer above with its reverse complement (5′-GACGUCCAUGUGCC-3′).^12^ (**C**) Enoxaparin: 16-mer low-molecular-weight heparin (~ 4.5 kDa).^19^ (**D**) P-PNA chimera: Peptide–peptide nucleic acid construct used in our previous work, Ac-EGEGEGGEggggEGGEGE(β-Ala)E (where “g” denotes PNA guanine units).^20^ (**E**) P0 used in this work Ac-EGEGEGGLLELALELLGGEGE(β-Ala)E-NH_2_. (**F**) P1: Fluorinated peptide analogue used in this work Ac-EGEGEGGLLEL-Phe(4-^19^F)-LELLGGEGE(β-Ala)E-NH₂ and (**G**) P2: Fluorinated peptide analogue used in this work Ac-GELEGLEGLEG-Phe(4-^19^F)-ELGLEGLE(β-Ala)EG-NH_2_.

Various polyanions have recently been proposed as possible ligands of the N-terminal region of the SARS-CoV-2 nucleocapsid protein^19–21^. These include enoxaparin^19,21^, a low molecular weight heparin also used in the treatment of severely ill COVID-19 patients, as well as an ad hoc designed chimeric molecule composed of a peptide nucleic acid core flanked by peptide termini^20^. The CSP analysis of ^1^H–^15^N HSQC titrations shows that, while all peptides perturb similar regions of the protein, these effects also significantly overlap with known RNA-binding sites, confirming the successful targeting achieved by the designed ligands (Fig. 12).

By analogy, this observation is also in line with previous studies showing that NTD(44–180) binds more effectively to single-stranded RNA fragments compared to their double-stranded counterparts^12^, highlighting a general preference of this region for ligands with greater conformational flexibility (Fig. 12).

Multidomain proteins, composed by alternating globular domains and intrinsically disordered regions, constitute an emerging field of research aimed at drug candidates discovery. Many proteins of biomedical interest share these properties, such as proteins involved in the onset of neurodegenerative diseases, cancer as well as infectious diseases^43–51^. In the presence of highly heterogenous target protein, well-established drug discovery strategies face significant limitations. To overcome these challenges, novel approaches should rely on the structural and dynamic characterization of the target protein by NMR.

The use of peptides represents a promising strategy to target highly dynamics proteins thanks to the versatility of the available building blocks comprising both natural as well as non-genetically encoded amino acids, and to the efficiency of modern synthetic methodologies^52^. Peptides can incorporate distinct functional modules, strategically distributed within a single primary sequence, enabling them to interact with multiple regions of the target protein, irrespective of its folded or intrinsically disordered nature. The combination of the different modules can also be used to increase the affinity and introduce selectivity. This strategy can be exploited to design diverse peptides that enable to experimentally investigate various features, such as the role of key amino acids, the importance of their distribution within the primary sequence as well as the presence of (or lack thereof) secondary structural elements.

To overcome possible limitations of *in-vivo* peptide proteolytic degradation, the introduction of non-natural residues can improve or overcome the pharmacokinetic and pharmacodynamic problems associated with all-natural peptides. Specifically, non-natural amino acids enhance proteolytic stability, leading to longer persistence in the body. Additionally, these unnatural moieties can finely tune the chemical properties required for biological activity.

Taken together, our findings provide a coherent framework for the rational design of peptide ligands targeting disordered or dynamic RNA-binding proteins. These results not only contribute to enhancing understanding of nucleocapsid–ligand interactions but also offer a foundation for the development of fluorinated peptides as selective molecular probes and possible inhibitors.

## Methods

### Solid phase peptide synthesis

#### Reagents

All Fmoc-protected amino acids, *N*,*N*′-diisopropylcarbodiimide (DIC), OxymaPure® (ethyl cyanohydroxyiminoacetate) were purchased from Iris Biotech GmbH (Marktredwitz, Germany). Tentagel® S RAM resin was purchased from Rapp Polymere (Tuebingen, Germany). Peptide-synthesis grade *N,N*-dimethylformamide (DMF) and acetonitrile (ACN) were purchased from Carlo Erba (Milano, Italy). Dichloromethane (DCM), trifluoroacetic acid (TFA), triisopropylsilane (TIS), acetic anhydride (Ac_2_O), and piperidine were purchased from Sigma-Aldrich (Milano, Italy).

#### Synthesis of P1 and P2

Both peptides P1 and P2 were synthesized by Induction-assisted Solid-Phase Peptide Synthesis (I-SPPS) following the Fmoc/*t*Bu orthogonal protection strategy, using the PurePep™Chorus™ automated peptide synthesizer (Gyros Protein Technologies, Uppsala, Sweden). Tentagel® S RAM resin was used (loading 0.23 mmol/g). The following Fmoc-amino acids were used: Fmoc-βAla-OH, Fmoc-L-Ala-OH, Fmoc-L-Glu(OtBu)-OH, Fmoc-L-Phe(4-F)-OH, Fmoc-Gly-OH, Fmoc-L-Leu-OH. Fmoc deprotections were performed with a solution of 20% piperidine in DMF for 60 s at 363 K. Peptide assembly was performed by repeating the SPPS coupling cycle for each amino acid, using Fmoc-protected amino acids (5 equivalents), OxymaPure® (5 equivalents), and DIC (5 equivalents) dissolved in DMF for 120 s at 363 K. The washing steps were performed using a mixture of AcOEt:DMSO 8:2 (v:v). All the Fmoc-L-Glu(OtBu)-OH and Fmoc-βAla-OH were coupled twice. *N*-acetylation was performed using a solution of 10% Ac_2_O in DMF for 5 min at 313 K for capping. Final cleavage and sidechain deprotections were performed using a cocktail mixture of TFA/TIS/H_2_O (95:2.5:2.5, v:v:v) at room temperature. After 2 h the resin was filtered off. The peptides P1 and P2 were precipitated with cold Et_2_O, centrifuged, and lyophilized. The crude peptides P1 and P2 were purified by Reverse-Phase Flash Liquid Chromatography on an Isolera One Flash Chromatography (Biotage, Uppsala, Sweden) using a SNAP Ultra C18 column (12 g) at 12 mL/min as solvent systems H_2_O (MilliQ) and ACN.

### Analytical characterization of the synthetic sequences P1 and P2

Analytical characterization of the peptides P1 and P2 was performed by HPLC using a Waters ACQUITY HPLC coupled to a single quadrupole ESI–MS (Waters® ZQ Detector, Waters Milford, MA, USA) supplied with a C18 column Supelco BIOshell A160 Peptide (100 × 3.0 mm, 2.7 μm) at 308 K, at 0.6 mL/min using solvent systems A (0.1% TFA in H_2_O) and B (0.1% TFA in ACN). The analytical data are reported in Table 1, while the chromatograms and mass spectrometry spectra are reported in the Supplementary Material (Figures S3–S4).

**Table 1:** Analytical characterization of the synthetic peptides. Eluents: 0.1% (v/v) TFA in H_2_O (A) and 0.1% (v/v) TFA in ACN (B), λ 215 nm. Gradient: 20–80 (% B) in 5 min; calculated as the ratio of obtained mass to theoretical mass. ESI–MS: detected as c[M^+2H^]^2+^.

Peptide	Sequence	R_t_ (min)^a^	HPLC purity(%)	Yield (%)^b^	ESI–MS (m/z) found (calcd)
P1	Ac-EGEGEGGLLEL-Phe(4-^19^F)-LELLGGEGE-βA-E-NH_2_	4.97	96	21	1205.5 (1205.3)
P2	Ac-GELEGLEGLEG- Phe(4-^19^F)-ELGLEGLE-βA-EG-NH_2_	4.08	97	18	1205.3 (1205.3)

### Circular dichroism

CD spectra of the peptides P1 and P2 were recorded using quartz cells of 1 cm path length with a JASCO J-1500 CD spectropolarimeter (Tokyo, Japan) at 298 K. The spectrum was measured in the 250 − 190 nm spectral range, 1 nm bandwidth, 3 accumulations, and 50 nm/min scanning speed. The peptides at 298 K. The spectrum was measured in the 250 − 190 nm spectral range, 1 nm bandwidth, 3 accumulations, and 50 nm/min scanning speed. The peptides P1 and P2 were dissolved in H_2_O and H_2_O:TFE 50:50 (v/v) at a concentration of 10 μM.

### Protein sample preparation

The NTD(44–180) and NTR(1–248) samples were prepared following previously reported protocols with a brief overview provided below^11,24,53^.

For the NTR(1–248) construct, the gene encoding the N protein fragment 1–248 was designed according to domain boundaries identified from the SARS-CoV homolog. A codon-optimized version of the gene was synthesized by Twist Bioscience and subcloned into the pET29b( +) vector using NdeI and XhoI restriction sites. The ^15^N-labeled NTR(1–248) protein was expressed in E. coli BL21 (DE3) using the Marley protocol. Cells were first cultured in 1 L Luria Bertani broth at 310 K until reaching an OD600 of 0.8. They were then transferred into 250 mL of isotopically labeled minimal medium containing 0.25 g/L ^15^NH_4_Cl (Cambridge Isotope Laboratories, Tewksbury, MA, USA). After 1-h period to clear unlabeled metabolites, protein expression was induced using 0.2 mM IPTG at 289 K for 18 h. Cells were harvested and stored overnight at 253 K.

The cell pellet was resuspended in 25 mM TRIS buffer (pH 8.0) with 1.0 M NaCl, 10% glycerol, and protease inhibitors (SIGMA). Following cell lysis by sonication, the lysate was clarified via centrifugation at 30,000 × g for 50 min at 277 K. The soluble protein fraction was dialyzed overnight against 25 mM TRIS buffer (pH 7.2) at 277 K. The dialyzed protein was applied to a 5 mL HiTrap SP FF column and eluted over 25 column volumes using a 70% linear gradient of 25 mM TRIS and 1.0 M NaCl. Protein-containing fractions were pooled and further purified using a HiLoad 16/1000 Superdex 75 pg column equilibrated with 25 mM potassium phosphate and 450 mM KCl at pH 6.5. Final concentration was achieved using centrifugal concentrators (10 kDa MWCO).

The NTD(44–180) sequence was derived from the SARS-CoV-2 NCBI reference genome entry NC_045512.2, which is identical to the GenBank entry MN908947. The gene, cloned into the pET28a( +) vector containing an N-terminal His6-tag and a tobacco etch virus (TEV) protease cleavage site, was generously provided by Prof. Fabio Almeida from the University of Rio de Janeiro. Following TEV-mediated proteolysis, the resulting 14.85 kDa protein was devoid of any artificial residues. Uniformly ^15^N-labeled NTD(44–180) was produced in *E. Coli* BL21 (DE3) cultured in M9 minimal medium supplemented with 1.0 g/L ammonium chloride (^15^NH_4_Cl) (Cambridge Isotope Laboratories, Tewksbury, MA, USA). Protein synthesis was triggered at an optical density of 0.7 at 600 nm (OD600) by adding 0.2 mM isopropyl-β-D-thiogalactopyranoside (IPTG), followed by incubation for 18 h at 289 K. The harvested cells were resuspended in 50 mM tris(hydroxymethyl)aminomethane (TRIS) at pH 8.0, 500 mM sodium chloride (NaCl), 20 mM imidazole, 10% v/v glycerol, and a protease inhibitor cocktail (SIGMAFAST). Cell disruption was performed via sonication, and the lysate was clarified by centrifugation at 30,000 × g for 30 min at 277 K. The clarified lysate was loaded onto a Ni(II)-NTA HisTrap HP column (GE Healthcare, Chicago, IL, USA), where the His6-Trx-tag was removed overnight at 277 K using a 1:10 v/v ratio of TEV protease to protein solution while dialyzing against a buffer containing 50 mM TRIS (pH 8.0), 500 mM NaCl, and 1 mM dithiothreitol (DTT). The TEV protease and the excised tag were subsequently eliminated using a second Ni(II)-NTA HisTrap HP purification step. The fractions containing the purified NTD(44–180) protein were identified via SDS-PAGE, pooled, and concentrated. Buffer exchange was conducted using either a PD-10 desalting column (GE Healthcare, Chicago, IL, USA) or dialysis, yielding a final buffer composition of 12.5 mM potassium phosphate (KH_2_PO_4_/K_2_HPO_4_), 50 mM potassium chloride (KCl), and 0.02% sodium azide (NaN_3_) at pH 6.5.

### NMR experiments

All the experiments for structural characterization of P1 and P2 were conducted at 298 K on a Bruker Avance NEO spectrometer equipped with a TCI probe optimized for ^1^H detection (900H) operating at 900.63 MHz ^1^H, 226.97 MHz ^13^C and 91.45 MHz ^15^N. In ^1^H-^1^H TOCSY and ^1^H-^1^H NOESY experiments, carrier frequency for ^1^H was set at 4.7 ppm. In ^1^H-^15^N HSQC^54^ carrier frequency for ^1^H was set at 4.7 ppm and for ^15^N at 117.0 ppm. Decoupling of ^15^N was achieved with garp4 composite pulse decoupling scheme with an RF field strength of 1.0 kHz. In ^1^H-^13^C HSQC^54,55^, carrier frequencies for ^1^H was set at 4.7 and for ^13^C at 45.0 ppm. Decoupling of ^13^C was carried out with bi_p5m4sp_4sp.2 super cycling decoupling sequence with an RF field strength of 4.5 kHz.

The titration of NTD(44–180) construct with P1 and P2 was carried out at 298 K using Bruker AVANCE NEO spectrometer operating at 600.03 MHz ^1^H, 564.64 MHz ^19^F, 150.79 MHz ^13^C, and 60.81 MHz ^15^N, equipped with a QCI-F probe (600F) optimized for ^1^H and ^19^F detection.

To follow the interaction between NTD(44–180) and the different peptides, ^1^H-^15^N HSQC in their sensitivity improvement fashion experiments^54^ were carried out. The carrier frequency of ^1^H was set at 4.7 while the carrier frequency of ^15^N was set at 117.0 ppm. Decoupling of ^15^N was achieved with garp4 composite pulse decoupling scheme with an RF field strength of 1 kHz. Other relevant acquisition parameters are reported in Table S11.

The titration of NTR(1–248) construct with P0 was carried out at 298 K using a Bruker Avance III spectrometer (Rheinstetten, Germany) equipped with a TCI probe optimized for ^1^H detection (950H) operating at 950.20 MHz ^1^H, 238.93 MHz ^13^C and 96.28 MHz ^15^N. In ^1^H-^15^N HSQC experiments, carrier frequency for ^1^H was set at 4.7 ppm and for ^15^N at 118.0 ppm. Decoupling of ^15^N was achieved with garp4 composite pulse decoupling scheme with an RF field strength of 1 kHz.

More acquisition parameters are reported in the Supplementary Materials (Table S11).

The dynamic properties characterization of P1 and P2 peptides in the different stages of the titration were monitored exploiting ^19^F CPMG, ^19^F Inversion Recovery and 1D ^19^F experiments carried out using the QCI-^19^F probe available at 600 MHz instrument^36,37,56,57^. The carrier frequency for ^19^F was set at − 116 ppm and for ^1^H at 7.0 ppm. ^1^H decoupling was achieved in all the experiments exploiting a garp4 composite pulse decoupling scheme with and RF field strength of 675 Hz. More acquisition parameters are reported in Supplementary Materials (Table S12).

#### Titration method

The interactions, which involved P1, P2, and NTD(44–180) were carried out using two 5 mm NMR tubes: a reference tube (tube 1) containing the protein in the NMR buffer (100 μM) and another tube (tube 2) with a batch of peptides P1 and P2 at 1.0 mM in the same buffer. Different aliquots of tube 2 were added into tube 1 in order to obtain the following protein:peptide molar ratio: 1:0.5, 1:1, 1:2, 1:4, and 1:8.

The interactions, which involved P0 and NTR(1–248) was carried out using a protein concentration of 70 μM by adding P0 in molar ratios of 1:0, 1:0.5, 1:1, 1:2., 1:4, 1:8.

### Chemical shift perturbations (CSP) analysis

At each step, 2D ^1^H-^15^N HSQC spectra were recorded to follow the interaction observing the Chemical Shift Perturbations (CSPs) relative to the protein residues affected by the peptide binding. This allowed to pinpoint the crucial region of the protein surface involved in the interaction. The titrations and the interactions between the protein constructs and the different ligands were followed observing the chemical shift perturbations for each residue of the target protein in both dimensions of NMR 2D ^1^H-^15^N HSQC correlation spectra (^1^H and ^15^N). The equation used to combine the CSP in the two dimensions of the nuclei is^58^:

1 $$CSP\left( {ppm} \right) = \sqrt {\left( {CSP\left( H \right) * CSP\left( H \right)} \right) + 0.097 * \left( {CSP\left( N \right) * CSP\left( N \right)} \right)}$$

where CSP is the difference in ppm between the observed chemical shift at different titration points and the chemical shift value of the reference spectrum. In particular, CSP(H) represents the CSP value in the proton dimension while CSP(N) the CSP value in the nitrogen dimension.

### Estimation of the K_d_

To calculate the *K*_*d*_ values, we selected the most perturbed residues, defined as those whose CSP values exceed the threshold of the mean plus one standard deviation, selecting isolated residues to ensure reliable fitting.

The dissociation constant (*K*_*d*_) for the interaction between the NTD(44–180) construct and the two different fluorinated ligands was determined through NMR spectroscopy measuring the CSP for each peak in a series of 2D ^1^H-^15^N HSQC spectra recorded at increasing concentrations of the ligand. The data were fitted using the following Equation^58^:

2 $$CSP\left( {ppm} \right) = \frac{{P + L + K - \sqrt {\left( {P + L + K} \right)^{2} - 4PL} }}{2P} \cdot M$$

with CSP previously defined, P is the total protein concentration, L is the peptide ligand concentration (P1 or P2) at different titration points, M represents the maximum expected CSP value upon the complete formation of the bound state, and *K* is the dissociation constant (*K*_*d*_). From the ligands point of view, the obtained CSP values from the 1D ^19^F spectra were used to determine the *K*_*d*_. Equation (3) was used for the fitting of *K*_*d*_ and it is reported below:

3 $$CSP\left( {ppm} \right) = \frac{{\left( {L + K + P} \right) - \sqrt {\left( {L + P + K} \right)^{2} - 4 \cdot P \cdot L} }}{2L} \cdot M$$

In both analysis we consider that the protein concentration remains constant throughout the whole titration as well as that the CSP value at concentration 0 of the peptide is 0. M, and K are the fitted values.

### Peptide-protein affinity simulations

#### Peptides building

Peptides P1 and P2 were built using the Build Structure module of the UCSF Chimera package^59^. Modified residues included β-alanine (PubChem CID: 239) and 4-fluoro-phenylalanine (PubChem CID: 4654). The resulting flat structures were energy minimized and MD simulated for 50 ns in AMBER03 ff. in GROMACS 2022.3-plumed_2.8.1 on NMRBox server^60,61^. The GMX cluster package was used to obtain the most predominant structure, prior to further analysis.

#### System preparation for replica exchange molecular dynamics

Two peptide systems (P1 and P2) were prepared using GROMACS, each one solvated in a cubic box with TIP3P water molecules and 0.15 M NaCl to ensure charge neutrality and approximate physiological ionic strength. The P1 system contained 316 peptide atoms, 3468 water molecules, and neutralizing ions, while P2 consisted of the same peptide sequence solvated with 2442 water molecules and 0.15 M NaCl.

### REMD simulations

REMD simulations were performed using GROMACS 2022.5 (Debian_2022.5_2) with OpenMP parallelization (GMX_OPENMP_MAX_THREADS = 128) on the CERM high-performance computing cluster. A total of 50 replicas were employed for P1 and 42 replicas for P2. Temperature distributions were generated using the Virtual Chemistry REMD Temperature Generator (https://virtualchemistry.org/remd-temperature-generator/) targeting an average exchange probability of ~ 0.2, with temperatures ranging from 298 to 460 K. Replica exchanges were attempted every 1000 steps (2 ps). Simulations were run using the leap-frog integrator with a timestep of 2 fs, totaling 200 ns per replica, corresponding to 10 microseconds of aggregate sampling for P1 and 8.4 microseconds for P2. Periodic boundary conditions were applied in all directions.

The temperature distributions for the replicas were as follows. For P1: 298.00, 300.79, 303.60, 306.43, 309.28, 312.16, 315.05, 317.96, 320.87, 323.83, 326.81, 329.81, 332.84, 335.88, 338.95, 342.04, 345.16, 348.30, 351.45, 354.64, 357.84, 361.07, 364.33, 367.60, 370.90, 374.23, 377.58, 380.96, 384.36, 387.78, 391.24, 394.72, 398.22, 401.75, 405.31, 408.89, 412.50, 416.13, 419.79, 423.48, 427.19, 430.94, 434.71, 438.51, 442.33, 446.19, 450.07, 453.98, 457.92, and 460.00 K. For P2: 298.00, 301.32, 304.67, 308.05, 311.46, 314.93, 318.39, 321.89, 325.42, 328.98, 332.57, 336.19, 339.85, 343.53, 347.25, 351.13, 354.91, 358.73, 362.58, 366.46, 370.38, 374.34, 378.33, 382.35, 386.40, 390.51, 394.64, 398.81, 403.02, 407.26, 411.54, 415.86, 420.21, 424.60, 429.03, 433.50, 438.01, 442.56, 447.15, 451.78, 456.45, and 460.00 K.

Long-range electrostatic interactions were treated with the Particle Mesh Ewald (PME) method with a real-space cutoff of 1.0 nm, and van der Waals interactions were also truncated at 1.0 nm. Temperature coupling was maintained via the V-rescale thermostat with separate coupling groups for the peptide and solvent (τ = 0.1 ps). Pressure was maintained at 1 bar using the Parrinello-Rahman barostat with isotropic coupling (τ = 2.0 ps). All bonds involving hydrogen atoms were constrained using the LINCS algorithm, allowing the use of a 2 fs timestep.

### Free energy landscape analysis

Following REMD simulations, trajectories from the reference (298 K) replica were analyzed to construct free energy landscapes (FELs). Principal Component Analysis (PCA) was performed on the covariance matrix of atomic positional fluctuations, focusing on the backbone atoms.

The first two principal components (PC1 and PC2) were used to project the conformational space, and the resulting distributions were converted into FELs using the Boltzmann relation:

$$\Delta G = - \kappa_{B} TlnP$$

where P is the probability density of observing a given conformation, *kB* is the Boltzmann constant, and T is the reference temperature (298 K). The FELs reveal the relative stability of conformational states, with free energy minima corresponding to the most populated and stable structures.

### Extraction of representative structures

Representative structures from each free energy basin were extracted by selecting the structures located at the global minima of the FEL. These conformations were further analyzed to characterize the folding pathways, secondary structure formation, and overall peptide stability.

### Peptide docking in NTD(44–180)

AutoDock Tools (ADT)^62^ was used to generate *.pdbqt files for both the peptides and the target protein, the N-terminal domain (NTD(44–180)) of the nucleocapsid (PDB ID: 6YI3). Preparation steps included the addition of polar hydrogens, assignment of Kollman charges, and redistribution of charges across the protein surface. The docking grid box was defined to encompass the whole protein (blind docking), and grid parameters were saved accordingly, although all peptides were docked in the RNA binding region. Molecular docking was performed using AutoDock Vina^59^, with an exhaustiveness setting of 8. For each peptide, docking poses were ranked according to their predicted binding free energy (ΔG), and the top ranked poses (most negative ΔG) were selected as starting configurations for MD simulations for detailed structural analyses. To ensure broader sampling of binding modes, eight distinct docking poses for each peptide were also used in individual MD simulations.

### Protein-peptide molecular dynamics

Molecular dynamics (MD) simulations were conducted using the PMEMD.CUDA module of AMBER^63^. The ff15ipq force field was employed due to its accurate handling of charged side chains and compatibility with β-alanine (Three-letters code: B3G). The non-standard residue 4-fluoro-phenylalanine (PFF) was parameterized using Antechamber (GAFF), with charges derived via RESP fitting at the HF/6-31G* level. The parameters were incorporated using pff.prepin and frcmod.pff.

Each peptide–protein complex was solvated in an octahedral box of TIP3P water molecules, with 0.15 M NaCl added and the system neutralized. The following equilibration steps were applied: a) energy minimization; b) heating from 100 to 300 K under constant volume (NVT); c) density equilibration under constant pressure (NPT). Production runs were carried out for 1 µs per system, using a 2 fs time step and SHAKE constraints on hydrogen atoms. Long-range electrostatics were treated with the Particle Mesh Ewald (PME) method. All simulations were performed on the CERM high-performance computing cluster. Binding free energy estimations were obtained using the MMPBSA.py script from AMBER Tools, considering 1000 evenly spaced frames from parts of each trajectory.

### Analysis of trajectories

Root means square deviation and fluctuation, hydrogen bonds, structures clusterization were analyzed using AMBER cpptraj. Trajectories were clustered using the DBSCAN algorithm implemented in cpptraj, based on backbone RMSD (atoms CA, C, N) of residues 44–180. Clustering was performed with neighborhood radius (ε) of 0.9 Å and a minimum of 25 frames, with random frame sieving (stride = 50) to reduce computational cost. Representative structures were extracted from the most populated clusters, and cluster populations and statistics were recorded for analysis.

## Supplementary Information

Below is the link to the electronic supplementary material.

Supplementary Material 1

## Data Availability

The data supporting the findings of this study are available within the paper and its Supplementary Information; if needed raw data will be made available upon request.
