# Supplementary material for: Peptide ligands to explore interactions with intrinsically disordered multidomain proteins: the case of SARS-CoV-2 nucleocapsid protein
Source: Sci Rep. 2026 Apr 18;16:18060. doi: 10.1038/s41598-026-46442-9 (PMC13254127; doi:10.1038/s41598-026-46442-9)
Supplement: Supplementary file 1 — Supplementary Material 1 [file 41598_2026_46442_MOESM1_ESM.docx]

**Peptide ligands to explore interactions with intrinsically disordered multidomain proteins: the case of SARS-CoV-2 nucleocapsid protein**

Tino A.S.^1,2^, Quagliata M.^1,3^, Schiavina M.^1,2^, Attanasio L.^1,2^, Santos B.P.O.^1,2^, Pacini L.^1,3^, Papini A.M.^1,3*^, Pierattelli R.^1,2*^, Felli I.C.^1,2*^

^1^ Department of Chemistry “Ugo Schiff”, University of Florence, Via della Lastruccia 3-13, 50019 Sesto Fiorentino, Florence, Italy

^2^ Magnetic Resonance Center (CERM), University of Florence, Via Luigi Sacconi 6, 50019 Sesto Fiorentino, Florence, Italy

^3^ Interdepartmental Research Unit of Peptide and Protein Chemistry and Biology (PeptLab), University of Florence, Via della Lastruccia 13, 50019 Sesto Fiorentino, Florence, Italy

*annamaria.papini@unifi.it; roberta.pierattelli@unifi.it; isabellacaterina.felli@unifi.it

**Supplementary material**


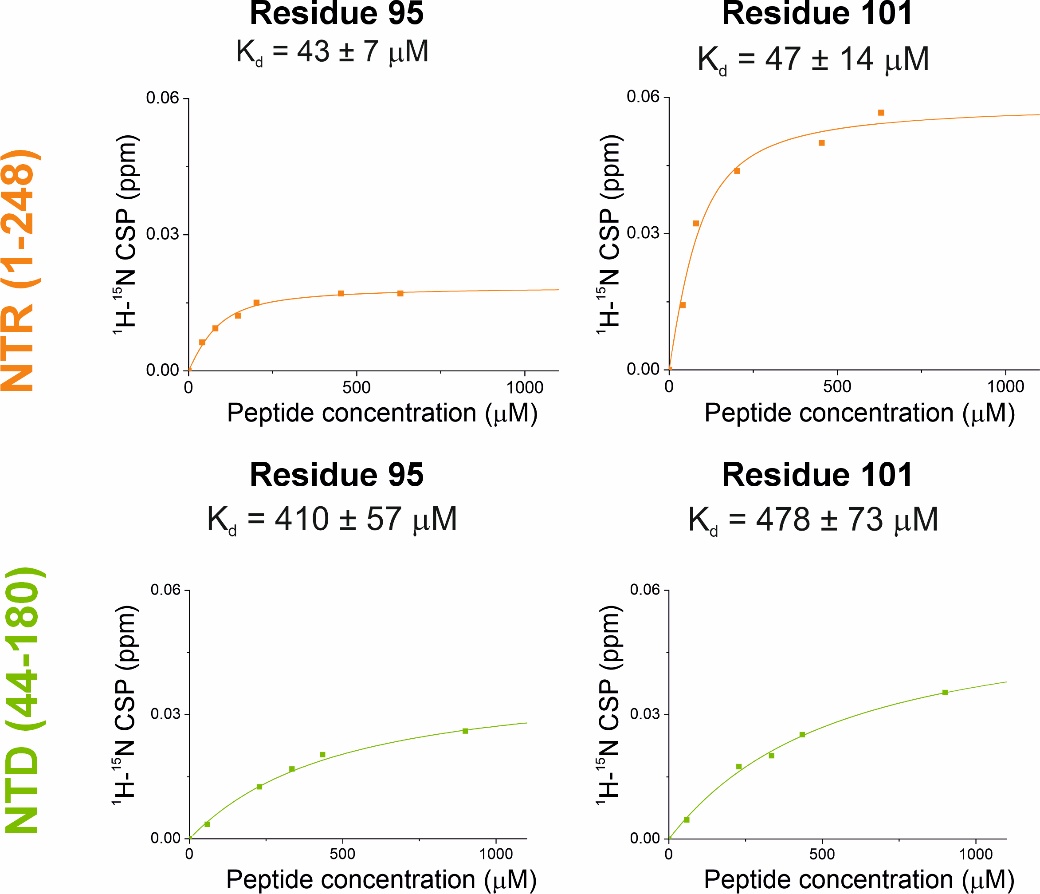


**Figure S1.** The figure shows the comparison of the fitting for the dissociation constants (*K_d_*) calculated for residues 95 and 101 in both titrations (NTR(1-248) top panels and NTD(44-180) bottom panels).


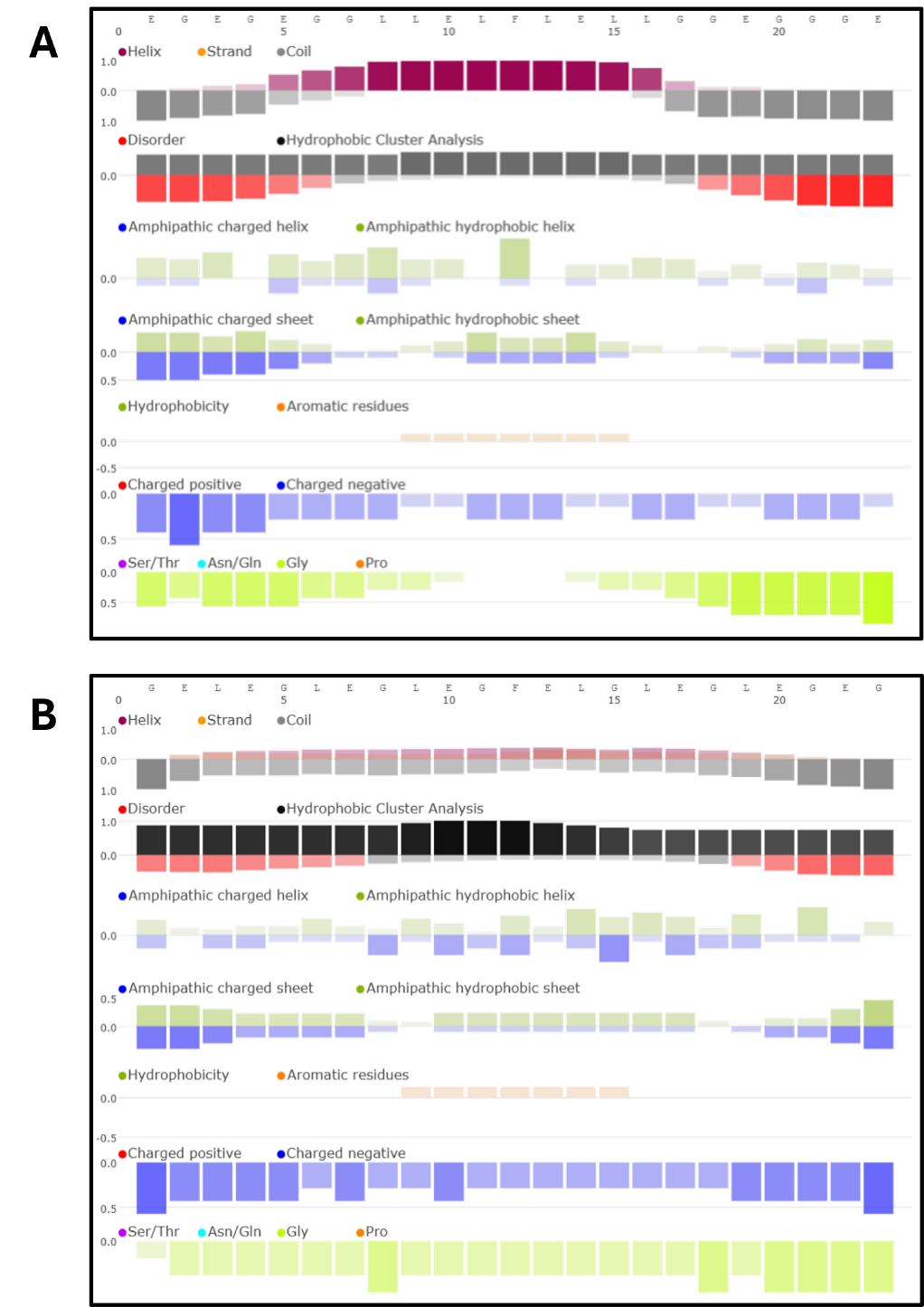


**Figure S2**. Panels A and B show the output of the FELLS software^1^ used to compute a variety of different features encoded in the peptides primary sequence. The FELLS plot reported are, from top to bottom of each panel, secondary structure prediction, intrinsic disorder predicted and hydrophobic clusters, amphipathic helices, amphipathic sheets, hydrophobicity and aromatic residues, charge clusters, special amino acid clusters. The sequence of each peptide is reported on top of each panel.

**Free energy profiles of peptides folding**


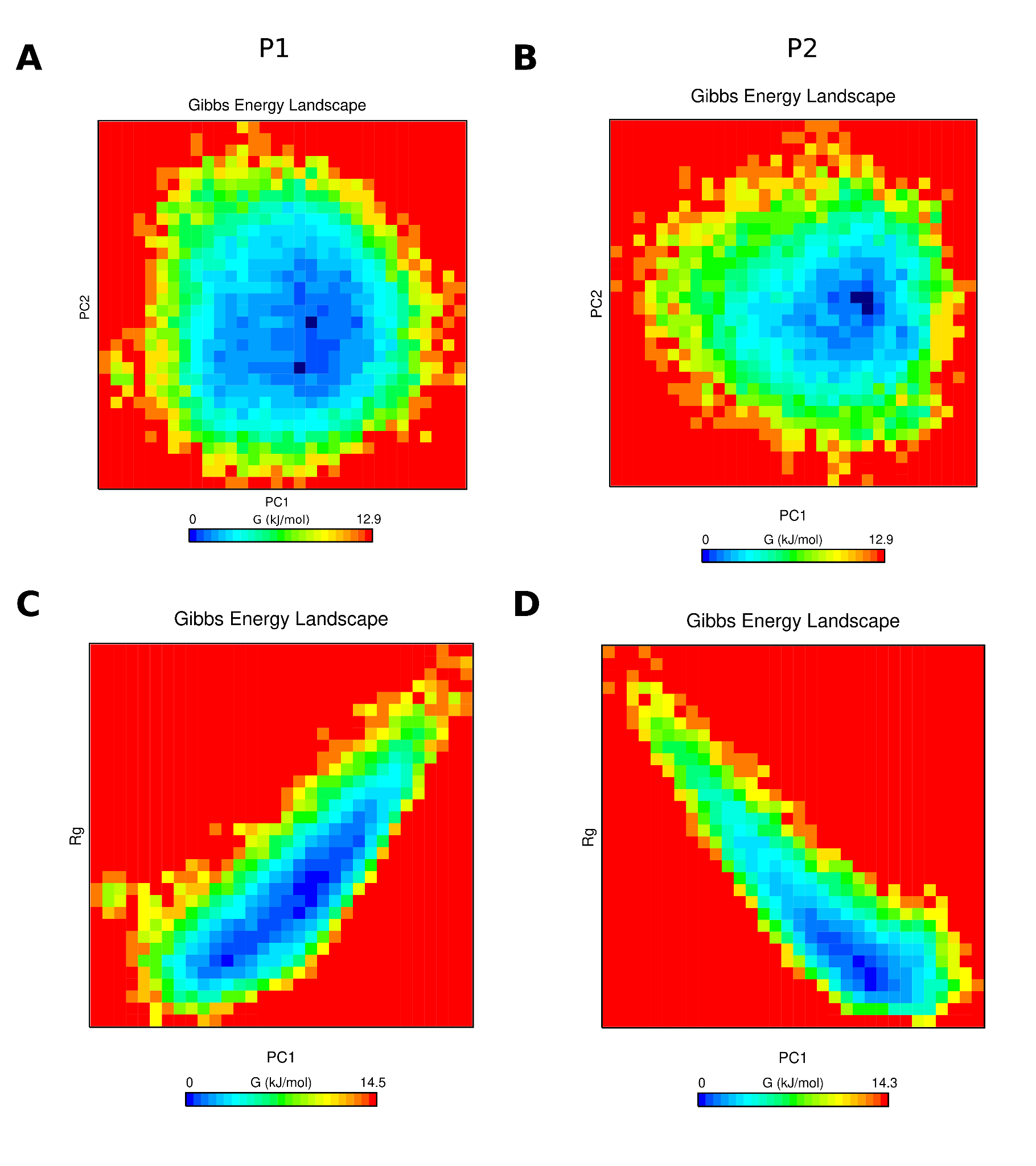


**Figure S3.** Comparative free energy landscapes of P1 and P2 along PC1, PC2 and Rg. (A) **P1** and (B) **P2** FELs in PC1-PC2 space. (C) **P1** and (D) **P2** FELs mapped as PC1 (x-axis) versus radius of gyration (Rg, y-axis). Color gradients reflect relative energy (red: high; dark blue: low).

**Analytical data of Peptides P1 and P2**

The analytical data, chromatograms, and mass spectrometry spectra related to the synthetic peptides are reported in the following **Figures S2–S3**

**Figure S4**. RP-UHPLC traces of peptide **P1**. Conditions: C18 column Supelco BIOshell A160 Peptide (100 x 3.0 mm, 2.7 μm); temperature, 308 K; flow, 0.6 mL/min; eluents, 0.1% (v/v) TFA in H_2_O (A) and 0.1% (v/v) TFA in ACN (B); λ, 215 nm; gradient, 20−80% B in 5 min. Rt = 4.97 min, **P1** (left panel). ESI-MS spectrum **P1**. ESI-MS (m/z): [M+2H]^2+^ 1205.5 (found), 1205.3 (calcd) (right panel).

**Figure S5**. RP-UHPLC traces of the peptide **P2**. Conditions: C18 column Supelco BIOshell A160 Peptide (100 x 3.0 mm, 2.7 μm); temperature, 308 K; flow, 0.6 mL/min; eluents, 0.1% (v/v) TFA in H_2_O (A) and 0.1% (v/v) TFA in ACN (B); λ, 215 nm; gradient, 20−80% B in 5 min. Rt = 4.08 min, **P2** (left panel). ESI-MS spectrum **P2**. ESI-MS (m/z): [M+2H]^2+^ 1205.3 (found), 1205.3 (calcd) (right panel).

**NMR characterization of peptides P1 and P2**

Characterization of **P1** started with the analysis of TOCSY and NOESY spectra. Focusing on the fingerprint region of TOCSY, we determined the peak patterns of the different residues (**Figure S6**), recognizing six sets of correlated cross peaks (peak arrays) that arise from Leu amino acids, eight sets deriving from Glu, eight sets from Gly, one for β-Ala, and one for Phe(4-^19^F). The sequence-specific assignment was performed employing TOCSY and NOESY spectra, through the following strategy: the striking example is the assignment of Leu13. In the Leu H^N^ region of the direct dimension of the NOESY spectrum, two unequivocal cross-peaks corresponding to the two ^1^H^B^ protons of the preceding Phe(4-^19^F)12 are observed. This confirms that the H^N^ peaks in question belong to Leu13. This process is repeated for each H^N^ resonance to achieve a sequence-specific assignment. 2D ^1^H-^15^N HSQC and ^1^H-^13^C HSQC were employed to focus on H^N^ and H^α^ protons, respectively, and were useful to assign ^15^N^H^ and ^13^C^α^. **Figure S7** reports the 2D ^1^H-^15^N HSQC employed for the assignment of H^N^ and ^15^N^H^. The resonance assignments of nuclear spins of **P1** obtained by NMR experiments are reported in **Table S1**.

**
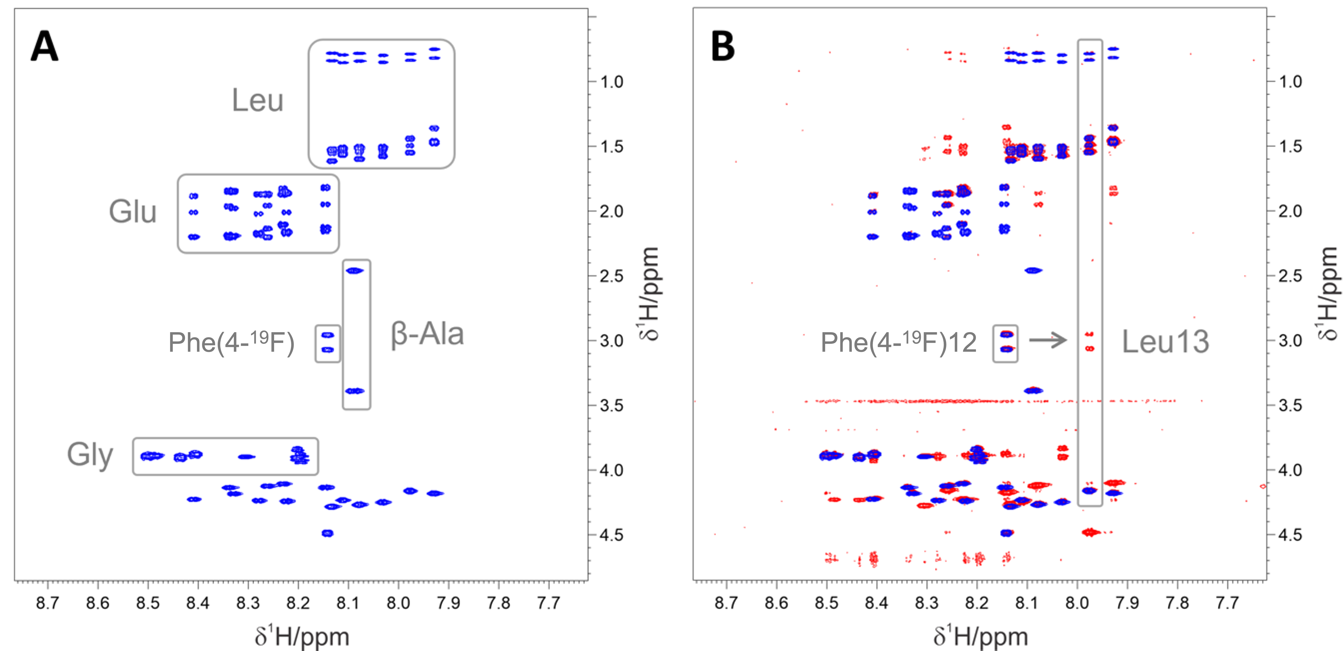
**

**Figure S6.** Representation of TOCSY (A) and a superimposition of TOCSY and NOESY (B) spectra of **P1**. The two panels show the region of the amide protons in the direct dimension and of aliphatic protons in the indirect dimension. The image also shows the first assignment done: Phe(4-^19^F)12 and Leu13. The spectra were acquired at 900 MHz on a 0.5 mM unlabeled sample in 12.5 mM phosphate buffer, 50 mM sodium chloride, pH 6.5 at 298K.


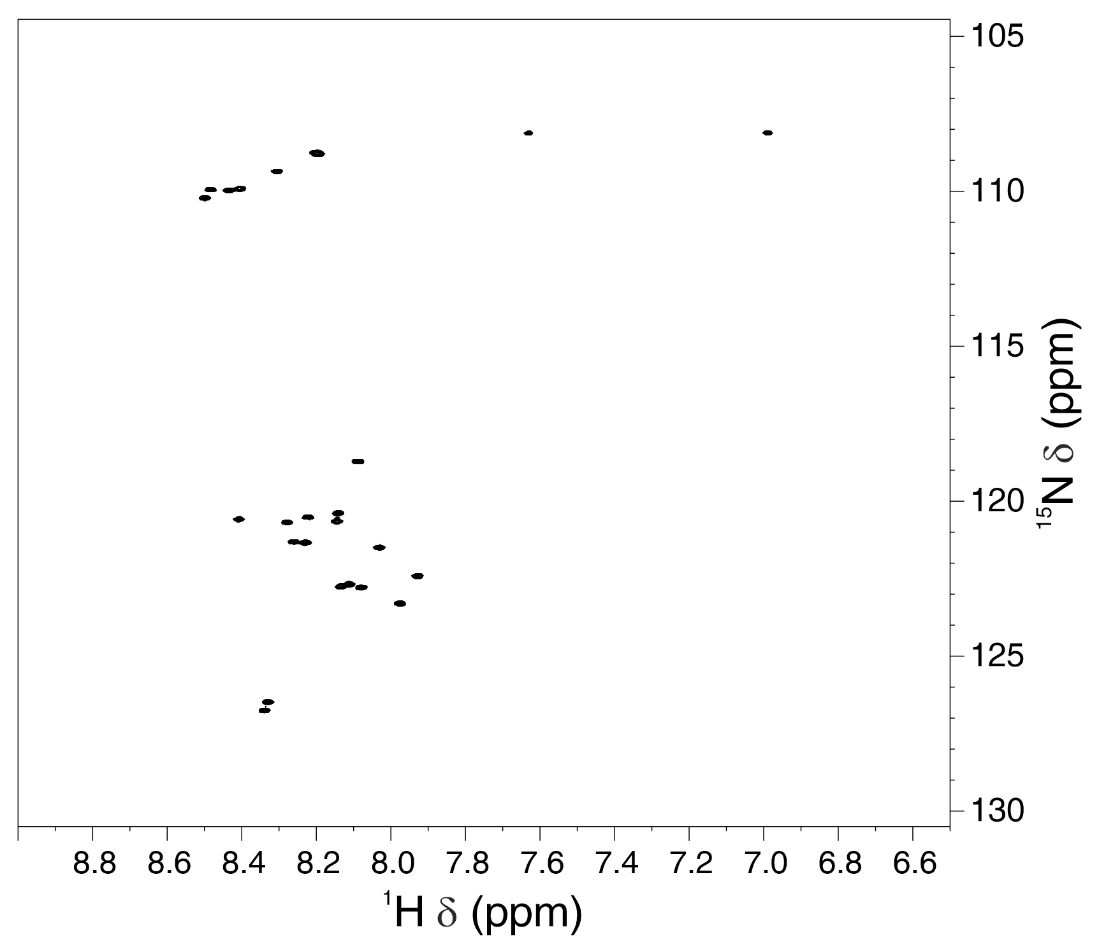
**Figure S7.** 2D ^1^H-^15^N HSQC spectrum of **P1** acquired at 900 MHz on a 0.5 mM unlabeled sample in 12.5 mM phosphate buffer, 50 mM sodium chloride, pH 6.5 at 298K.

**Table S1.** Assignments of **P1** by ^1^H-^1^H NOESY, ^1^H-^1^H TOCSY, ^1^H-^15^N HSQC, and ^1^H-^13^C HSQC experiments.

| **Residue name** | **Residue number** | **HA1 and HA2 (ppm)** | **HB2 and HB3 (ppm)** | | **H sidechain (ppm)** | | **HN (ppm)** | | **N (ppm)** | | **CA (ppm)** | | |
| --- | --- | --- | --- | --- | --- | --- | --- | --- | --- | --- | --- | --- | --- |
| Glu | 1 | 4.27 | 2.06  2.93 | | 2.28 | | 8.41 | | 126.60 | | 56.81 | | |
| Gly | 2 | 3.98 |  | |  | | 8.58 | | 110.30 | | 45.37 | | |
| Glu | 3 | 4.31 | 2.10 | | 2.26 | | 8.36 | | 120.79 | | 56.74 | | |
| Gly | 4 | 3.97 |  | |  | | 8.57 | | 110.06 | | 45.48 | | |
| Glu | 5 | 4.21 | 1.96  2.04 | | 2.29  2.23 | | 8.31 | | 120.64 | | 56.78 | | |
| Gly | 6 | 3.99 |  | |  | | 8.52 | | 110.05 | | 45.22 | | |
| Gly | 7 | 3.99  3.92 |  | |  | | 8.29 | | 108.86 | | 45.28 | | |
| Leu | 8 | 4.33 | 1.66  1.62 | | 1.62  0.93  0.88 | | 8.11 | | 121.61 | | 55.42 | | |
| Leu | 9 | 4.32 | 1.65  1.63 | | 1.62  0.93  0.87 | | 8.19 | | 122.78 | | 55.49 | | |
| Glu | 10 | 4.32 | 1.95  1.93 | | 2.25  2.10 | | 8.31 | | 121.46 | | 56.68 | | |
| Leu | 11 | 4.26 | 1.56  1.44 | | 1.53  0.90  0.83 | | 8.01 | | 122.52 | | 55.33 | | |
| Phe(4-^19^F) | 12 | 4.57 | 3.04  3.15 | |  | | 8.23 | | 120.49 | | 57.85 | | |
| Leu | 13 | 4.24 | 1.63  1.58 | | 1.57  0.91  0.87 | | 8.06 | | 123.42 | | 55.46 | | |
| Glu | 14 | 4.19 | 1.95  1.91 | | 2.19 | | 8.34 | | 121.40 | | 56.85 | | |
| Leu | 15 | 4.35 | 1.68  1.63 | | 1.62  0.92  0.86 | | 8.16 | | 122.88 | | 55.16 | | |
| Leu | 16 | 4.36 | 1.69  1.62 | | 1.64  0.92  0.86 | | 8.22 | | 122.89 | | 55.22 | | |
| Gly | 17 | 3.98 |  | |  | | 8.39 | | 109.45 | | 45.30 | | |
| Gly | 18 | 4.02  3.97 |  | |  | | 8.28 | | 108.87 | | 45.16 | | |
| Glu | 19 | 4.31 | 2.09  1.97 | | 2.28 | | 8.49 | | 120.70 | | 56.83 | | |
| Gly | 20 | 3.96 |  | |  | | 8.49 | | 110.00 | | 45.22 | | |
| Glu | 21 | 4.22 | 2.03  1.90 | | 2.23  2.20 | | 8.23 | | 120.76 | | 56.48 | | |
| β-Ala | 22 | 4.22 | 2.05  1.93 | |  | | 8.17 | | 118.83 | | 38.49 | | |
| Glu | 23 | 4.22 | 2.05  1.93 | | 2.27 | | 8.42 | | 126.87 | | 56.68 | | |
|  |  |  | |  | |  | |  | |  | |  |  |

**P2** was characterized following the same procedure as **P1** as shown in **Figure S8-S9** and in **Table S2**.


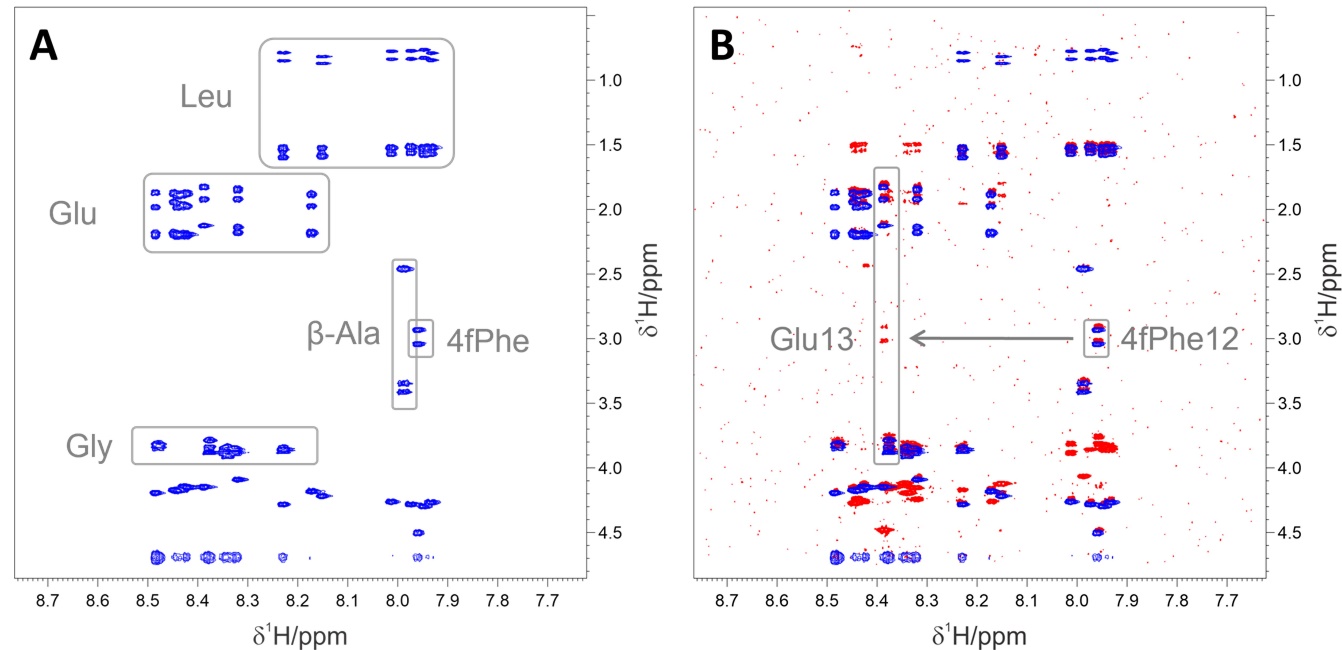


**Figure S8.** ^1^H-^1^H TOCSY (A) and a superimposition of ^1^H-^1^H TOCSY and ^1^H-^1^H NOESY (B) spectra of **P2**. The two panels show a zoom in the region of the amide protons in the direct dimension and the one of the aliphatic protons in the indirect dimension. The image also shows an example of assignment: 4F-Phe12 to Glu13. The spectra were acquired at 900 MHz on a 0.5 mM unlabeled **P2** sample in 12.5 mM phosphate buffer, 50 mM sodium chloride, pH 6.5 at 298K.


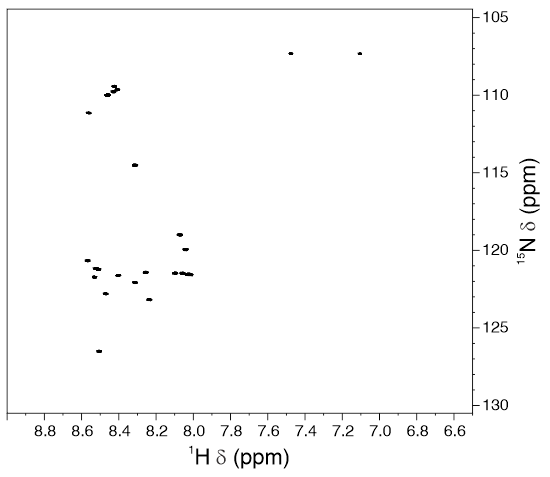
**Figure S9.** 2D ^1^H-^15^N HSQC spectrum of **P2** acquired at 900 MHz on a 0.5 mM unlabeled sample in 12.5 mM phosphate buffer, 50 mM sodium chloride, pH 6.5 at 298K.

**Table S2.** Assignments of **P2** by ^1^H-^1^H NOESY, ^1^H-^1^H TOCSY, ^1^H-^15^N HSQC, and ^1^H-^13^C HSQC experiments.

| **Residue number** | **Residue name** | **HA1 and HA2 (ppm)** | **HB2 and HB3 (ppm)** | **H sidechain (ppm)** | **HN (ppm)** | **N (ppm)** | **CA (ppm)** |
| --- | --- | --- | --- | --- | --- | --- | --- |
| Gly | 1 | 3.91  3.90 |  |  | 8.56 | 111.12 | 44.98 |
| Glu | 2 | 4.28 | 2.06  1.96 | 2.28 | 8.57 | 120.66 | 56.92 |
| Leu | 3 | 4.37 | 1.67  1.60 | 1.61  0.87 | 8.31 | 122.07 | 55.09 |
| Glu | 4 | 4.27 | 2.05  1.96 | 2.26 | 8.25 | 121.41 | 56.96 |
| Gly | 5 | 3.97  3.96 |  |  | 8.46 | 109.98 | 45.50 |
| Leu | 6 | 4.36 | 1.64  1.61 | 1.59  0.91  0.86 | 8.06 | 121.46 | 55.13 |
| Glu | 7 | 4.22 | 2.05  1.96 | 2.27 | 8.51 | 121.21 | 56.54 |
| Gly | 8 | 3.98  3.93 |  |  | 8.43 | 109.75 | 45.35 |
| Leu | 9 | 4.38 | 1.66  1.61 | 1.60  0.90  0,85 | 8.03 | 121.55 | 55.10 |
| Glu | 10 | 4.25 | 2.02  1.96 | 2.26 | 8.53 | 121.71 | 57.08 |
| Gly | 11 | 3.92  3.86 |  |  | 8.45 | 109.48 | 45.33 |
| Phe(4-^19^F) | 12 | 4.59 | 3.01  3.12 |  | 8.04 | 119.93 | 57.80 |
| Glu | 13 | 4.23 | 2.00  1.91 | 2.20 | 8.47 | 122.79 | 57.06 |
| Leu | 14 | 4.29 | 1.67 | 1.62  0.95  0.89 | 8.23 | 123.18 | 55.43 |
| Gly | 15 | 3.99  3.91 |  |  | 8.42 | 109.40 | 45.42 |
| Leu | 16 | 4.34 | 1.65  1.62 | 1.60  0.92  0.86 | 8.09 | 121.45 | 55.23 |
| Glu | 17 | 4.26 | 2.06  1.98 | 2.29 | 8.52 | 121.17 | 56.93 |
| Gly | 18 | 3.96  3.94 |  |  | 8.41 | 109.64 | 45.42 |
| Leu | 19 | 4.34 | 1.65  1.62 | 1.60  0.93  0.87 | 8.02 | 121.55 | 55.17 |
| Glu | 20 | 4.17 | 2.00  1.93 |  | 8.40 | 121.61 | 56.93 |
| β-Ala | 21 | 3.46 | 2.05  1.95 |  | 8.07 | 119.01 | 38.43 |
| Glu | 22 | 4.24 | 2.05  1.95 | 2.28 | 8.51 | 126.49 | 57.07 |
| Gly | 23 | 3.96  3.92 |  |  | 8.31 | 114.50 | 45.38 |

***K_d_* determination**


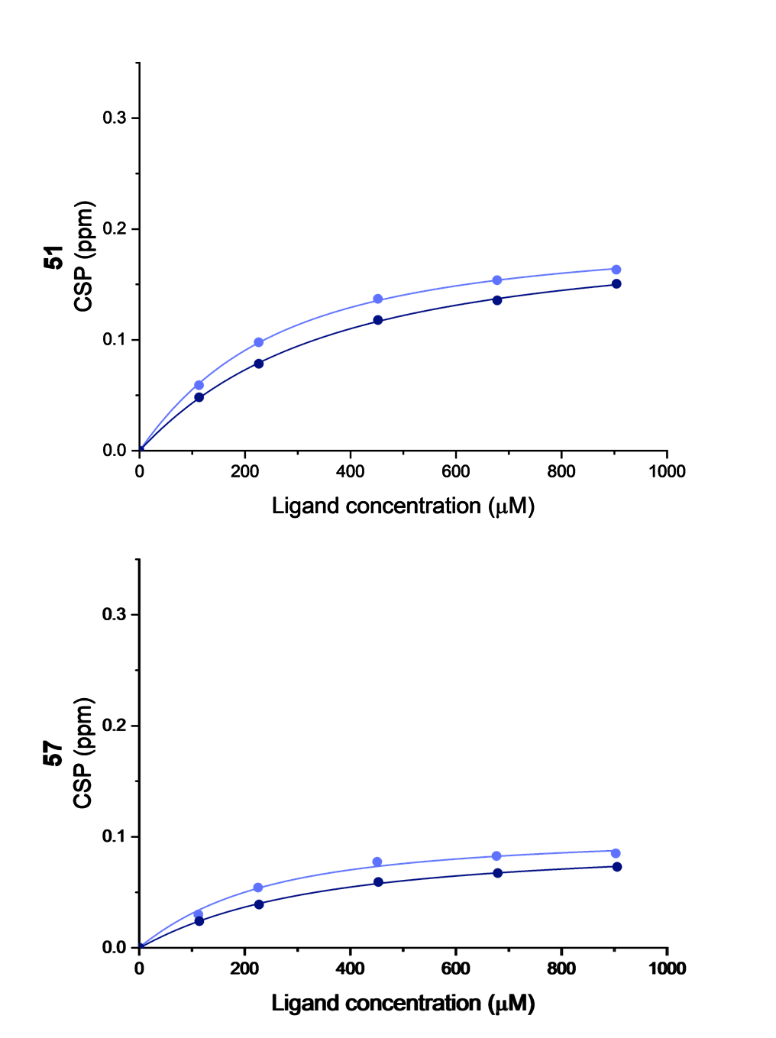

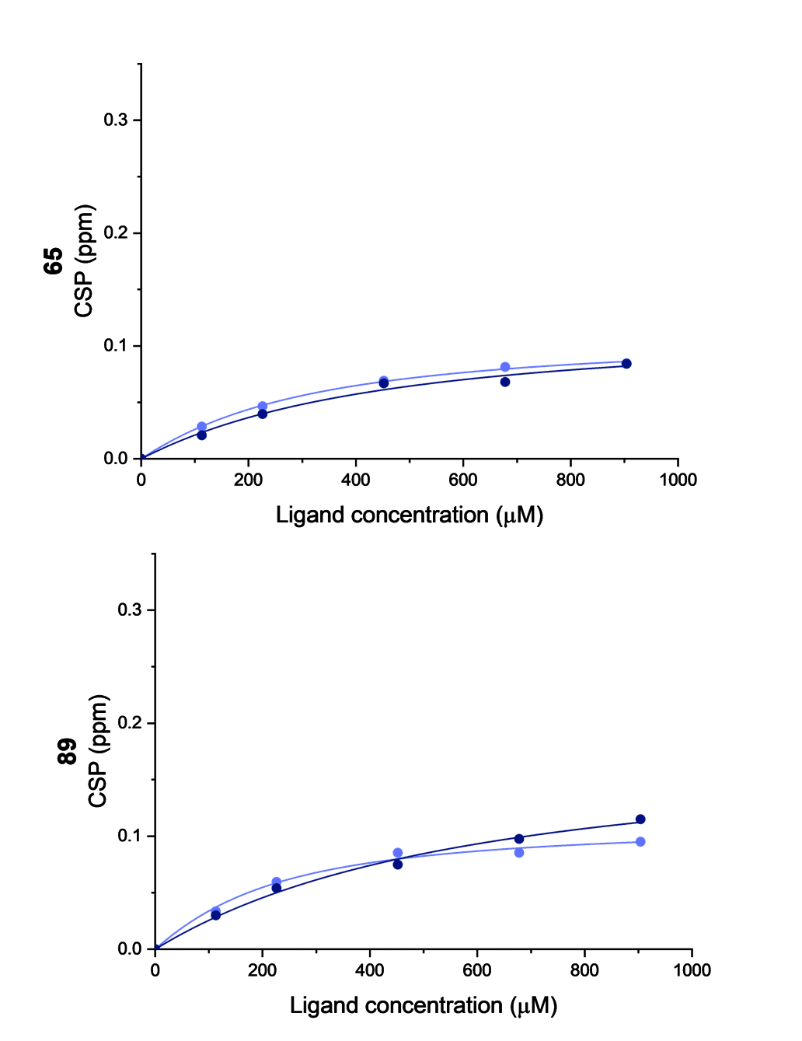

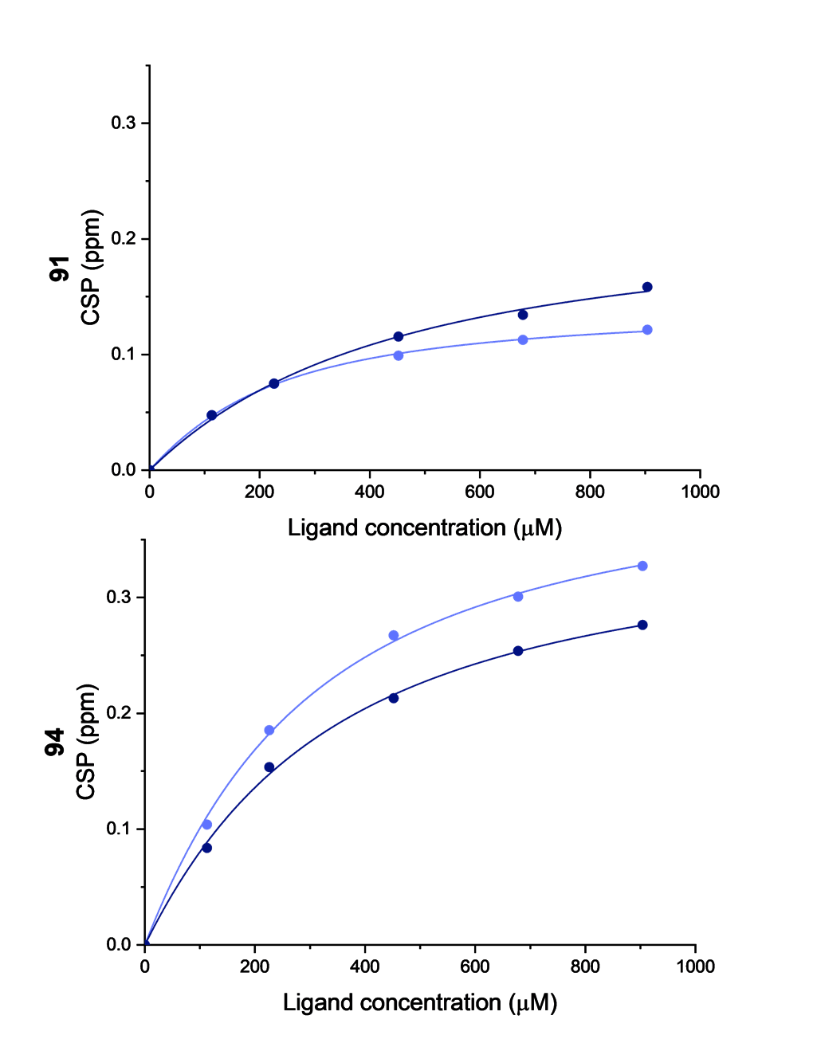

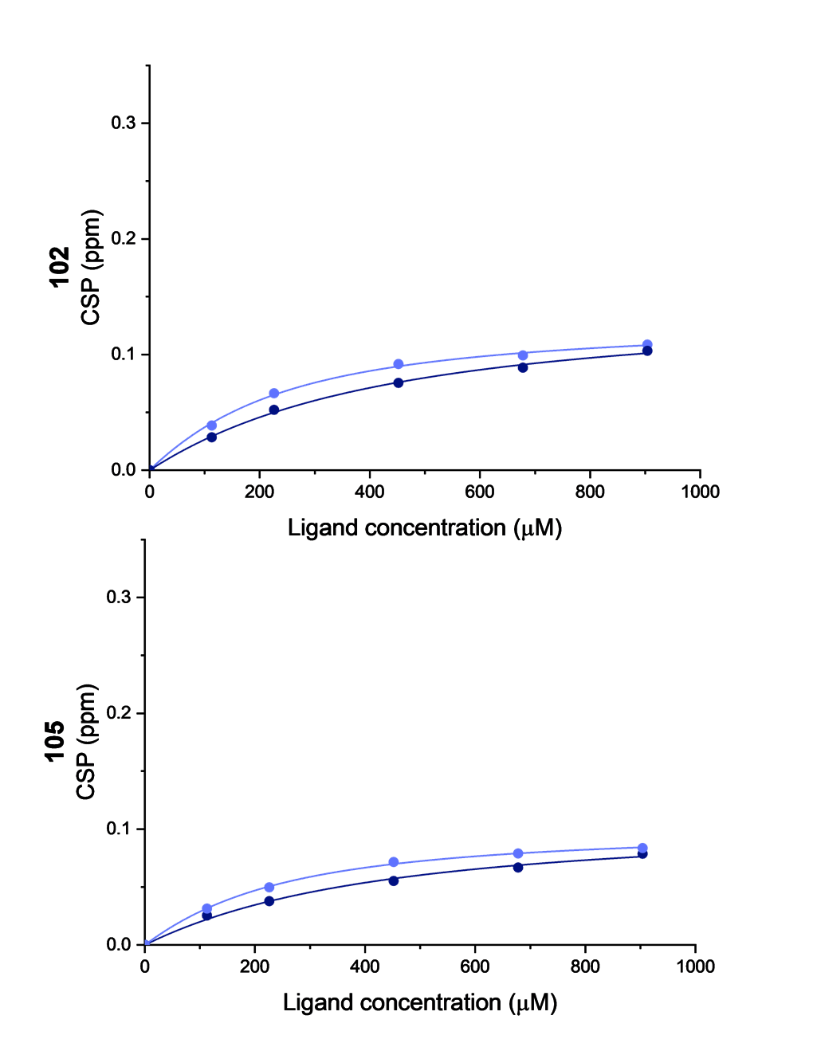

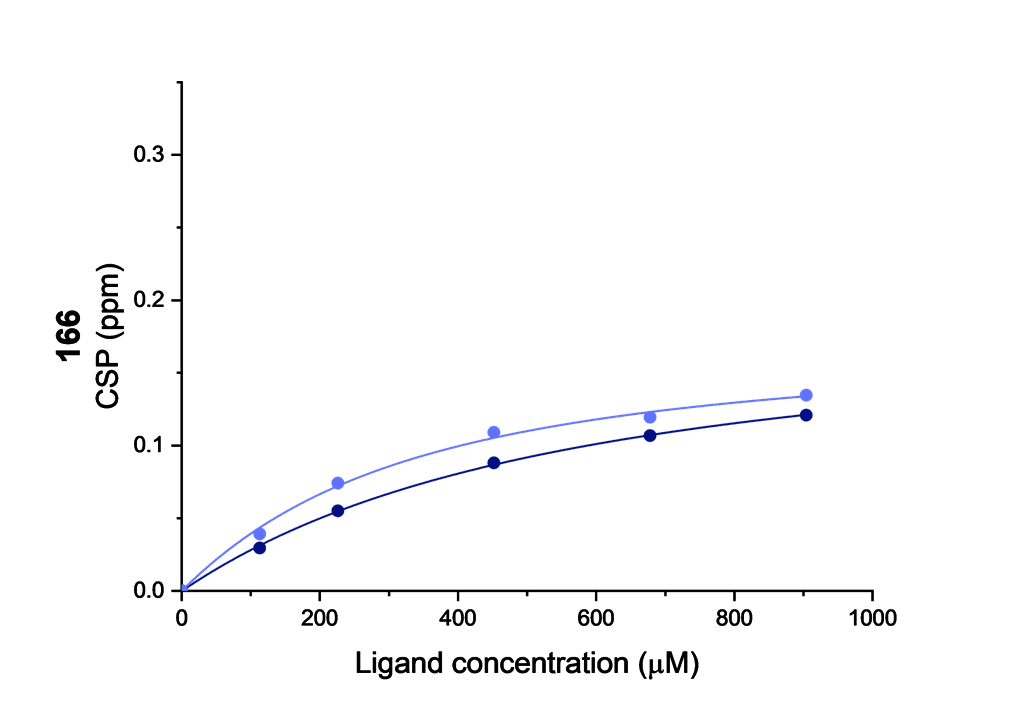

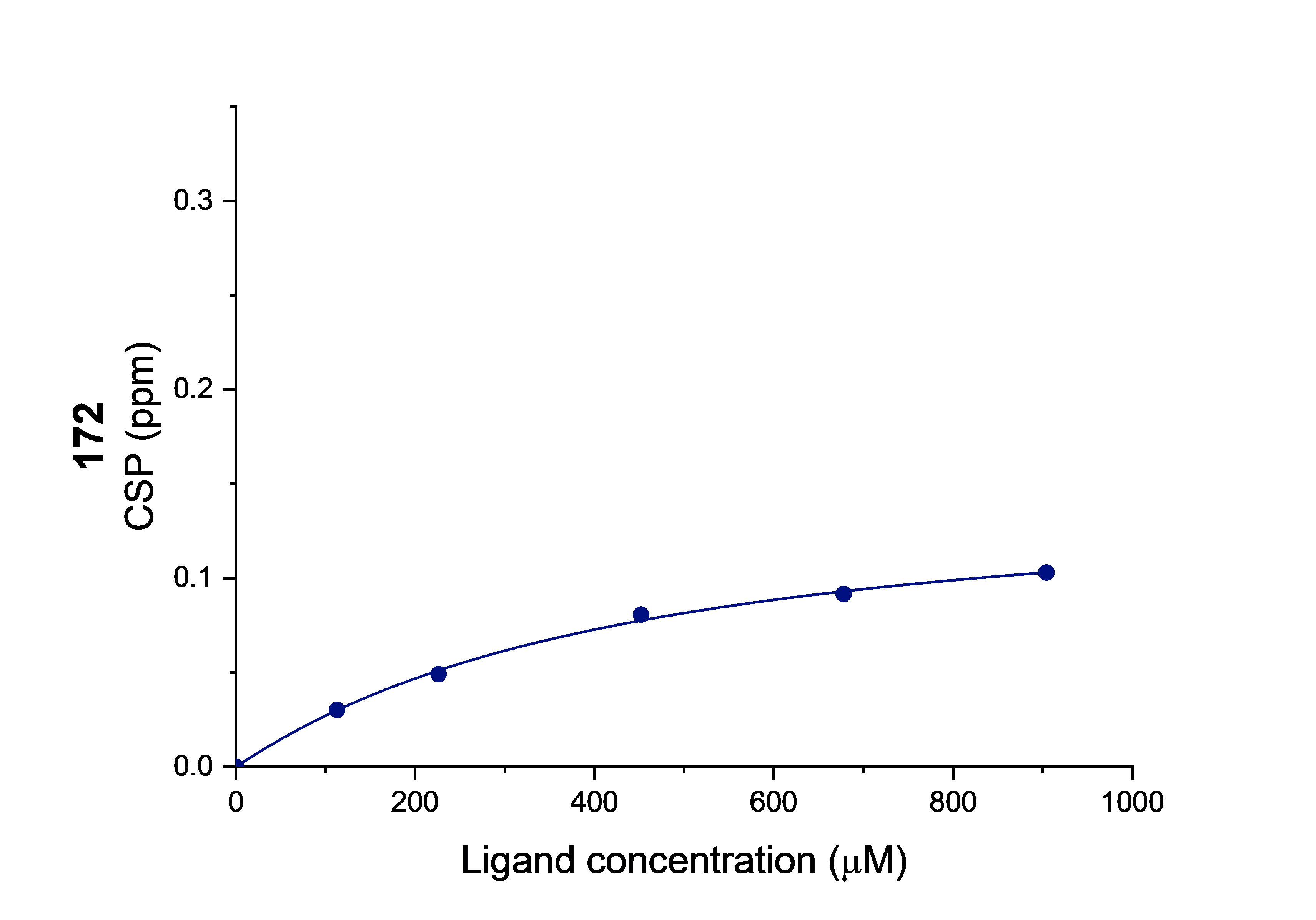

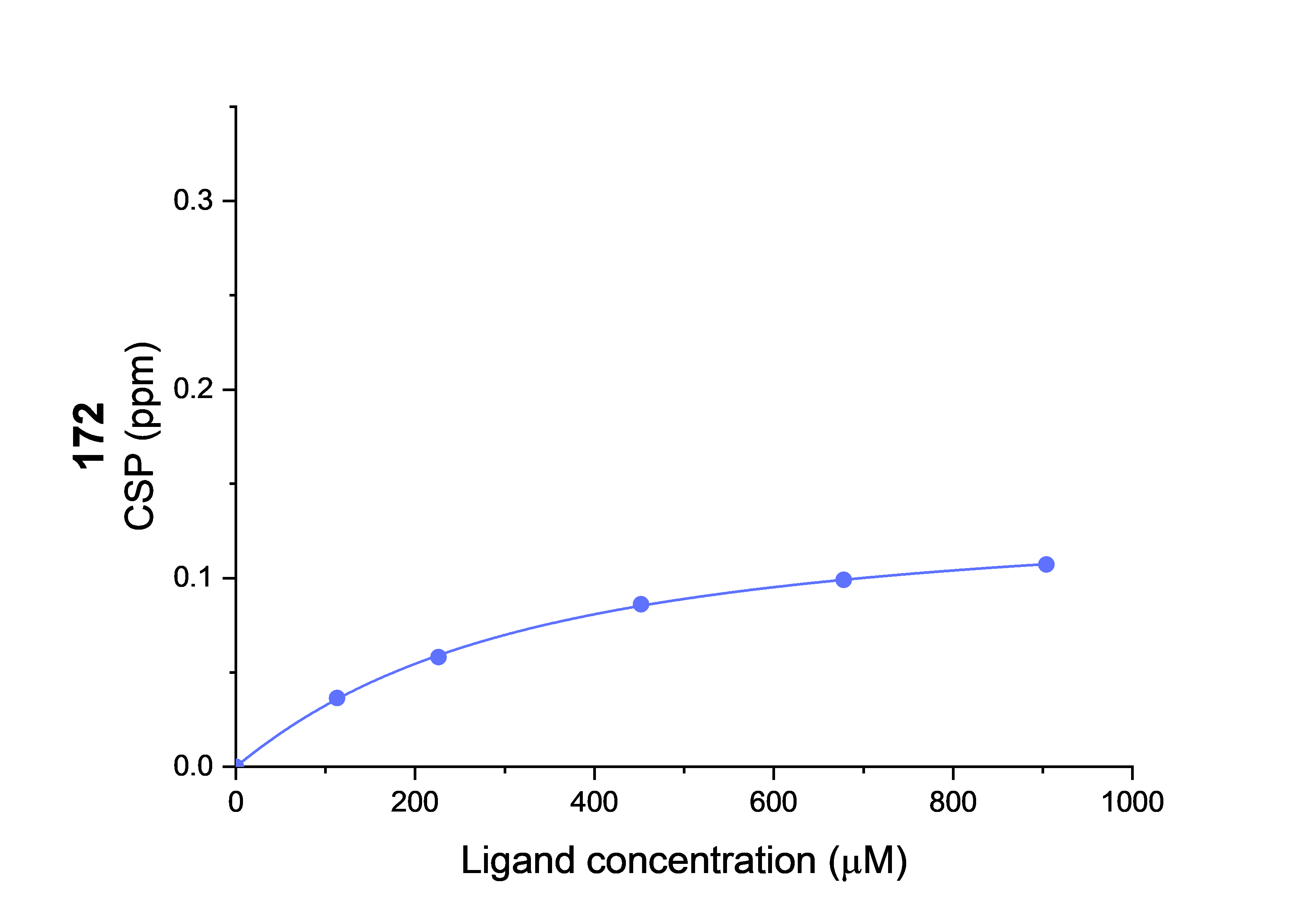


**Figure S10.** Fittings of the measured CSP values upon addition of increasing amounts of peptide **P1** (blue) and **P2** (light blue**)** are reported for the most affected residues (S51, T57, K65, R89, T91, I94, K102, S105, T166, and Y172) together with the *K_d_* estimated using equation 2 (In the main text).

**Table S3.** *K_d_* values for the most perturbed residues. All the *K_d_* are expressed in μM. Errors on the *K_d_* as obtained from the fitting procedure are reported in round bracket.

| **Res** | **P0 *K_d_* (error)** | **P1 *K_d_* (error)** | **P2 *K_d_* (error)** |
| --- | --- | --- | --- |
| SER 51 | 769 (±156) | 277 (±11) | 182 (±7) |
| THR 57 | // | 251 (±14) | 160 (±32) |
| LYS 65 | // | 375 (±111) | 242 (±22) |
| ARG 89 | 571 (±170) | 509 (±98) | 154 (±36) |
| THR 91 | 966 (±324) | 368 (±47) | 157 (±9) |
| ILE 94 | 846 (±222) | 268 (±21) | 232 (±20) |
| LYS 102 | 508 (±83) | 354 (±40) | 170 (±18) |
| SER 105 | // | 353 (±64) | 175 (±12) |
| THR 166 | 1622 (±523) | 484 (±27) | 259 (±40) |
| TYR 172 | 249 (±62) | 349 (±37) | 241 (±9) |

**1D ^19^**
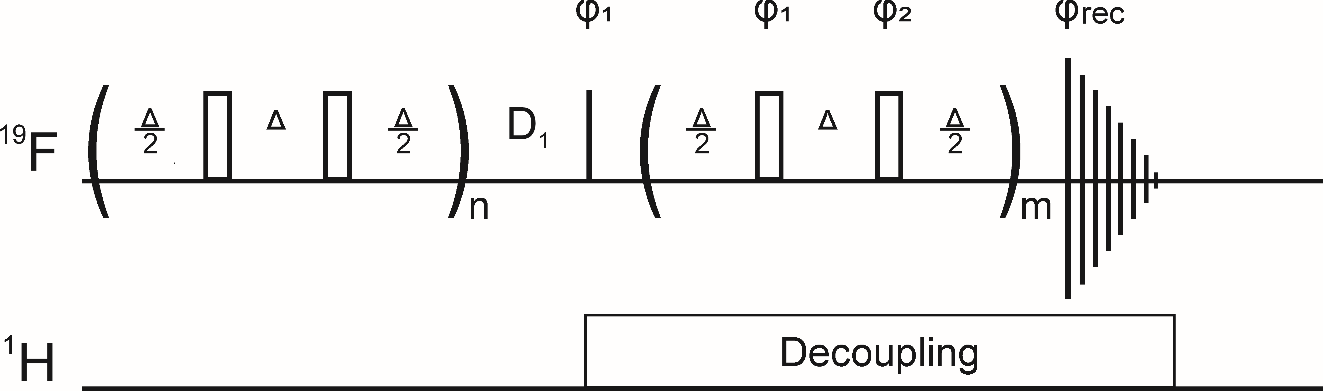
**F and CPMG experiments**

**Figure S11.** Schematic representation of the ¹⁹F CPMG pulse sequence. n = loop max – m, with n ranging from 1 to loop max.
φ₁ = x –x –x x y –y –y y; φ₂ = –x x x –x –y y y –y; φ_rec = x –x –x x y –y –y y. This pulse sequence, which includes ^1^H decoupling implemented with the ^19^F-QCI probe, constitutes a minor modification of ^2,3^.

**Table S4.** Measured *T_2_, R_2_, d7* delay, and number of repetitions *(n*) of CPMG block set (with a duration of 4∙d7) for experiments with free **P1**, NTD(44-180) + **P1** (1:1), and NTD(44-180) + **P1** (1:8). Errors as obtained from the fitting are reported in square bracket.

|  | ***T_2_*** (s) [error] | ***R_2_*** (s^-1^) [error] | ***d7*** (ms) | ***N*** |
| --- | --- | --- | --- | --- |
| **P1** | 0.190 [±8.78∙10^-3^] | 5.26 [±0.243] | 20 | from 1 to 10 |
| **NTD(44-180) + 8 eq P1** | 0.0515 [±1.24∙10^-3^] | 19.4 [±0.473] | 4.0 | from 1 to 6 |
| **NTD(44-180) + 1 eq P1** | 0.0395 [±1.18∙10^-3^] | 25.3 [±0.761] | 4.0 | from 1 to 6 |

**Table S5.** Measured *T_2_, R_2_, d7* delay and number of application *(n*) of CPMG block set for experiments on **P2** free, NTD(44-180) + **P2** (1:1), and NTD(44-180) + **P2** (1:8). Errors as obtained from the fitting are reported in square bracket.

|  | ***T_2_*** (s) | ***R_2_*** (s^-1^) | ***d7*** (ms) | | ***N*** |
| --- | --- | --- | --- | --- | --- |
| **P2** | 0.189 [±2.57∙10^-3^] | 5.27 [±7.18∙10^-2^] | 12 | from 1 to 10 | |
| **NTD(44-180) + 8 eq P2** | 0.102 [±1.49∙10^-3^] | 9.73 [±1.43∙10^-1^] | 3.0 | from 1 to 10 | |
| **NTD(44-180) + 1 eq P2** | 0.066 [±1.15∙10^-3^] | 15.2 [±2.67∙10^-1^] | 3.0 | from 1 to 6 | |

**Inversion Recovery experiments**
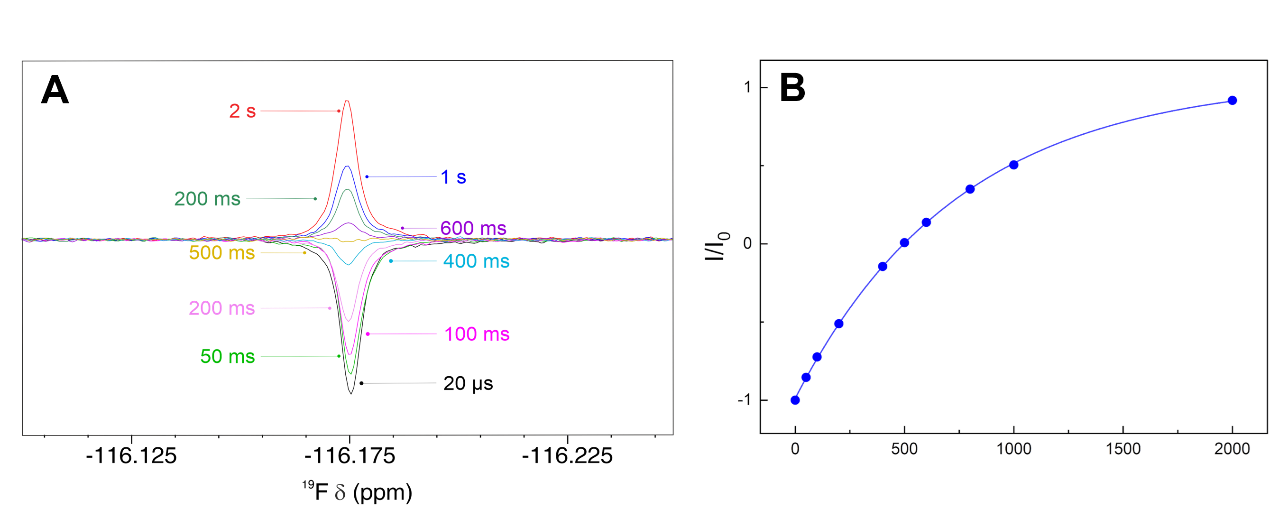


**Figure S12.** (A) ^19^F Inversion Recovery experiments were performed on a solution of **P1**; an ^1^H decoupling pulse was applied during acquisition. Delays were set to span the following range: 20 μs, 50 ms, 100 ms, 200 ms, 400 ms, 500 ms, 600 ms, 800 ms, 1 s, and 2 s. (B) Plot of the intensity of the ^19^F peak from each experiment vs. the corresponding relaxation delay
(intensity is normalized to the initial magnetization, I0), T1 = 752 ± 9.61 ms.

Inversion Recovery with ^1^H decoupling during acquisition was also performed on samples containing **P1** in the presence of the NTD(44-180) protein construct, using 1:1 and 1:8 NTD(44-180)-to-Peptide ratios. *T_1_* measured values are reported in **Table S6**, and the superimposition of the three obtained plots is shown in **Figure S13**.

**Table S6.** Measured *T_1_* and *R_1_* for i) **P1** free, ii) NTD(44-180) + **P1** (1:1), and iii) NTD(44-180) + **P1** (1:8). The reported values were obtained performing ^19^F Inversion recovery with ^1^H-^19^F decoupling during acquisition. Errors as obtained from the fitting are reported in square bracket.

|  | ***T_1_*** (s) | ***R_1_*** (s^-1^) |
| --- | --- | --- |
| **P1** | 0.752 [± 9.60∙10^-3^] | 1.33 [± 1.70∙10^-2^] |
| **NTD(44-180) + 8 eq P1** | 0.725[± 2.05∙10^-2^] | 1.38 [± 3.91∙10^-2^] |
| **NTD(44-180) + 1 eq P1** | 0.730[± 2.83∙10^-2^] | 1.37 [± 5.27∙10^-2^] |


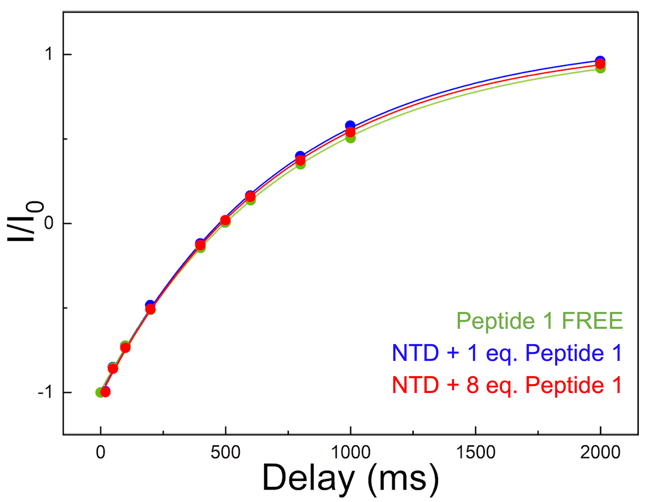
**Figure S13.** Superimposition of ^19^F Inversion Recovery plots of **P1** (green), NTD(44-180) and **P1** (1:1 ratio, blue), NTD(44-180) and **P1** (1:8 ratio, red). Delays were set to span the following range: 20 μs, 50 ms, 100 ms, 200 ms, 400 ms, 500 ms, 600 ms, 800 ms, 1 s, and 2 s. ^1^H-^19^F decoupling pulse was applied during acquisition (intensity is normalized to the initial magnetization, *I_0_*).

These sets of experiments show very little differences in terms of T_1_.


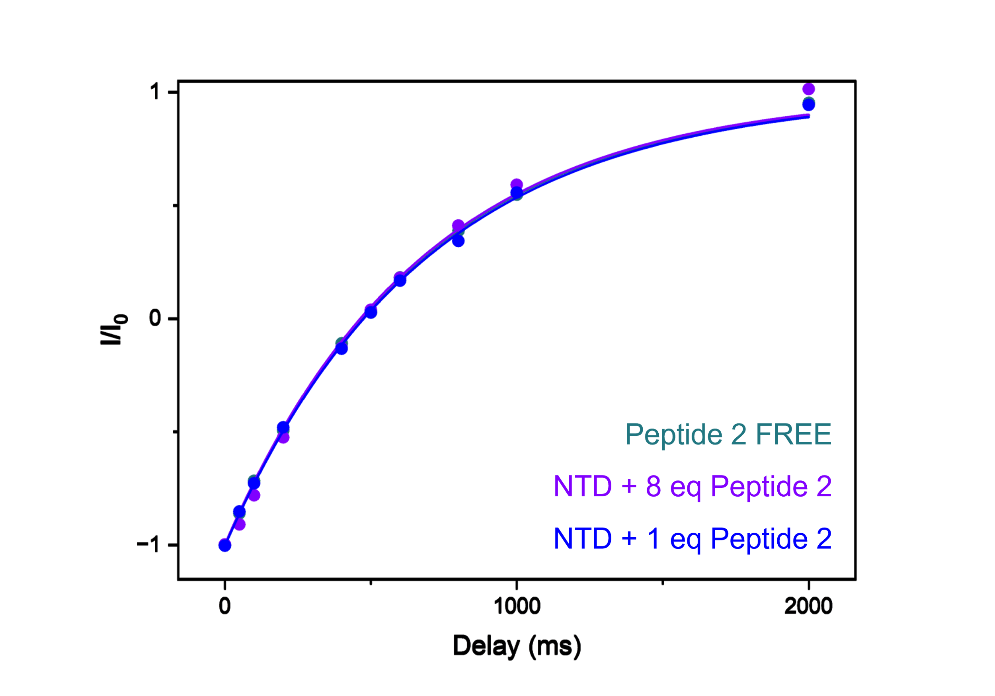


**Figure S14.** Superimposition of ^19^F Inversion Recovery plots of **P2** (deep aqua green), NTD(44-180) and **P2** in 1:1 ratio (blue), NTD(44-180) and **P2** in 1:8 ratio (purple). Delays were set to span the following range: 20 μs, 50 ms, 100 ms, 200 ms, 400 ms, 500 ms, 600 ms, 800 ms, 1 s, and 2 s. ^1^H-^19^F decoupling pulse was applied during acquisition (intensity is normalized to the initial magnetization, *I_0_*).

**Table S7.** Measured *T_1_*, and *R_1_* for **P2** free, NTD(44-180) + **P2** (1:1), and NTD(44-180) + **P2** (1:8). The reported values were obtained performing ^19^F Inversion recovery with ^1^H-^19^F decoupling during acquisition. Errors as obtained from the fitting are reported in square bracket.

|  | ***T_1_*** (s) | ***R_1_*** (s^-1^) |
| --- | --- | --- |
| **P2** | 0.676[± 8.18∙10^-3^] | 1.48 [± 1.79∙10^-2^] |
| **NTD(44-180) + 8 eq P2** | 0.671[± 2.07∙10^-2^] | 1.49[± 4.60∙10^-2^] |
| **NTD(44-180) + 1 eq P2** | 0.684[± 9.89∙10^-3^] | 1.46 [± 2.11∙10^-2^] |

The docking conformations used as start positions for the molecular dynamics are displayed in **Figure** **S15-S16**.

**Docking and molecular dynamics (MD) simulations**


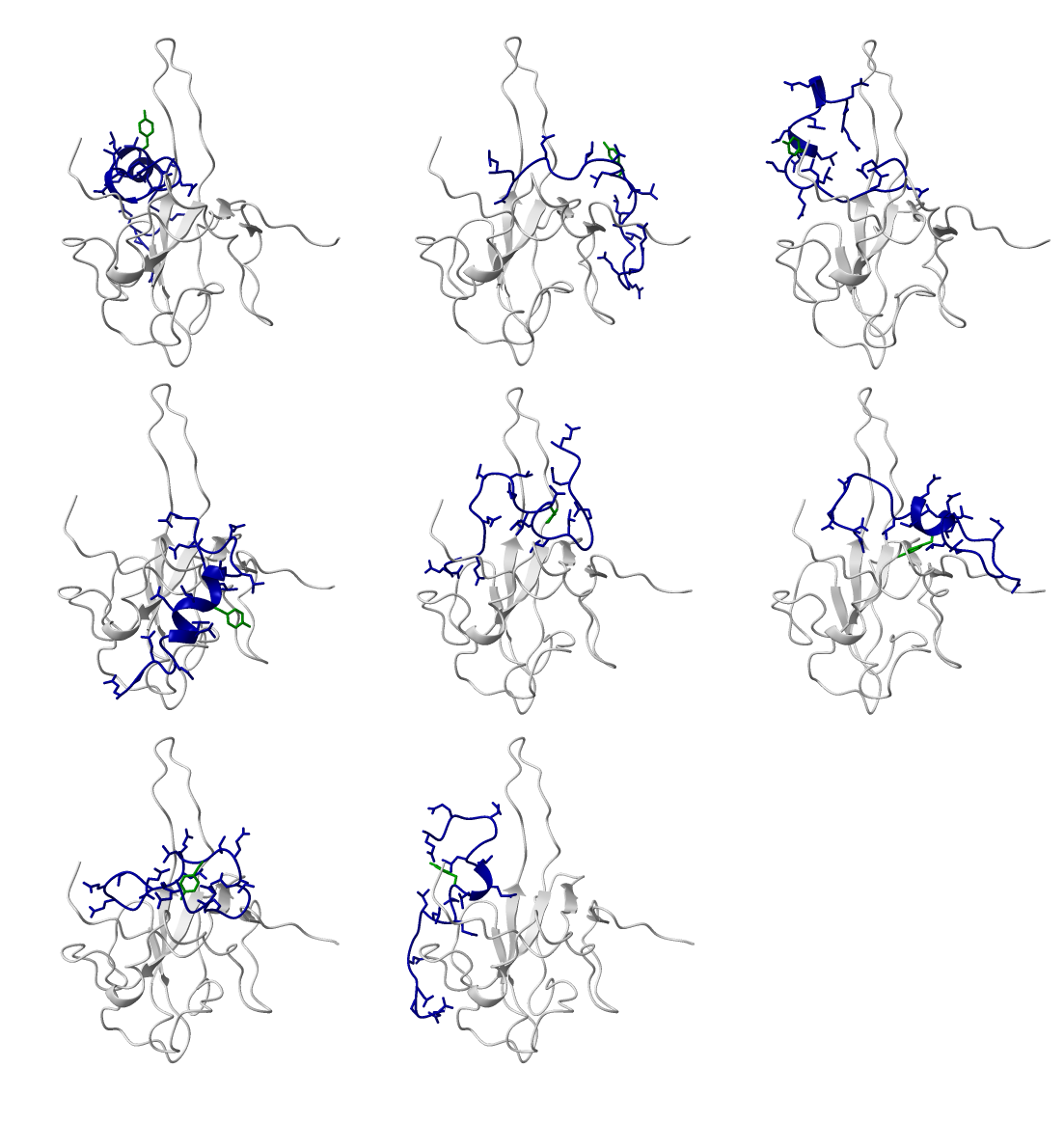


**Figure S15.** NTD(44-180)-**P1** docking positions. These conformations were used as start positions for MD. NTD(44-180) in grey and **P1** in blue.


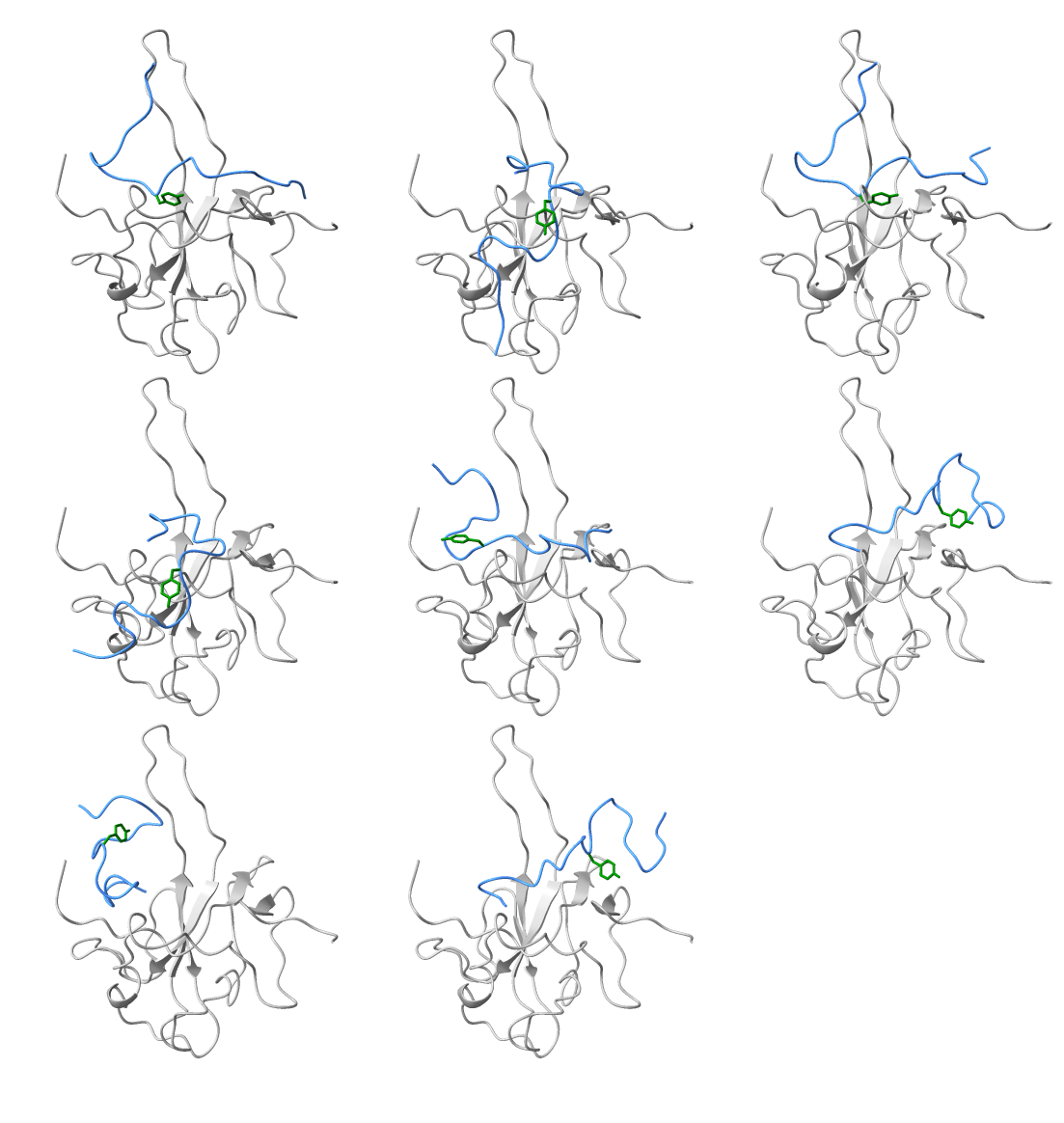


**Figure S16.** NTD(44-180)-**P2** docking positions. Eight conformations were used as start positions for MD. NTD(44-180) in gray and **P2** in light blue.

**Table S8. Clustering analysis of MD trajectories using DBSCAN based on backbone RMSD.**

Clustering of the MD trajectories resulted in multiple conformational states, with the most populated clusters representing a small fraction of the total ensemble, reflecting the high flexibility of the system. The first and second clusters correspond to the most populated conformations and were used for structural analysis. Trajectories were clustered using DBSCAN in cpptraj (ε = 0.9 Å, minPts = 25; stride 50) based on backbone RMSD (C^α^, C, N atoms) of residues 44–180. Clusters are characterized by the number of frames, population (%), average intra-cluster RMSD (Å), and standard deviation (Å) for each peptide system.

|  | **Cluster** | **Frames** | **Population (%)** | **Average Distance (Å)** | **Standard Deviation (Å)** |
| --- | --- | --- | --- | --- | --- |
| **P1** | 1 | 30646 | 6.10 | 1.32 | 0.29 |
|  | 2 | 24718 | 4.90 | 1.26 | 0.25 |
|  | 3 | 15246 | 3.00 | 1.14 | 0.18 |
|  | 4 | 11945 | 2.40 | 1.15 | 0.22 |
|  | 5 | 9552 | 1.90 | 1.09 | 0.19 |
|  | 6 | 8037 | 1.60 | 1.06 | 0.17 |
|  | 7 | 6118 | 1.20 | 1.01 | 0.14 |
|  | 8 | 3918 | 0.80 | 0.96 | 0.13 |
|  | 9 | 3095 | 0.60 | 0.96 | 0.14 |
|  | 10 | 2905 | 0.60 | 0.96 | 0.13 |
| **P2** | 1 | 15222 | 3.00 | 1.07 | 0.17 |
|  | 2 | 5117 | 1.00 | 1.06 | 0.16 |
|  | 3 | 4354 | 0.90 | 1.04 | 0.13 |
|  | 4 | 4041 | 0.80 | 0.99 | 0.12 |
|  | 5 | 3522 | 0.70 | 1.04 | 0.13 |
|  | 6 | 2859 | 0.60 | 0.99 | 0.12 |
|  | 7 | 2665 | 0.50 | 1.01 | 0.14 |
|  | 8 | 2358 | 0.50 | 0.95 | 0.13 |
|  | 9 | 2222 | 0.40 | 0.97 | 0.12 |
|  | 10 | 2205 | 0.40 | 1.00 | 0.13 |


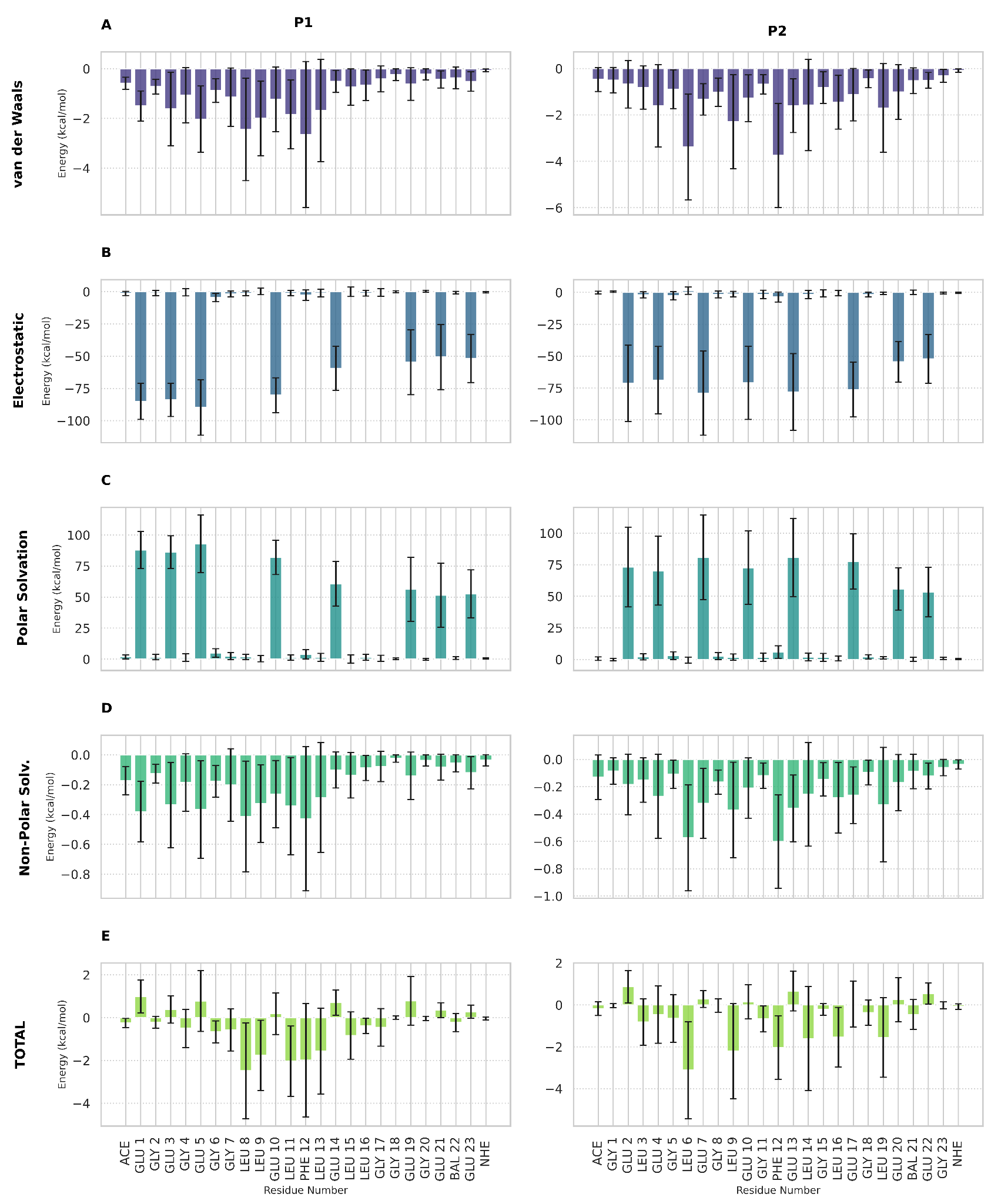


**Figure S17.** Comparison of interaction energy components between **P1** and **P2** and the target NTD(44-180) protein during MD simulations**.** (A) van der Waals interactions, (B) electrostatic interactions, (C) polar solvation energy, (D) non-polar solvation energy, and (E) total binding energy. Energy components were computed via MM/PBSA. Differences highlight the contribution of each term to the overall binding affinity of the peptides. It can be observed that the electrostatic and polar solvation contributions mainly derive from the Glu residues in both sequences. The most pronounced van der Waals contacts involve Phe12 for **P1** and **P2**, although Leu6 has a similar relevance in **P2**. The ΔG per residue is also shown in panel E of Figure S17. For **P1**, the residues with lowest energy range from Leu8 to Leu13 (the folded core of **P1**) with slightly lower contribution from the other residues. On the other hand, the residues with the lowest energy on **P2** are more evenly distributed: Leu6, Leu9, Phe12, Leu14, Leu16, and Leu19.


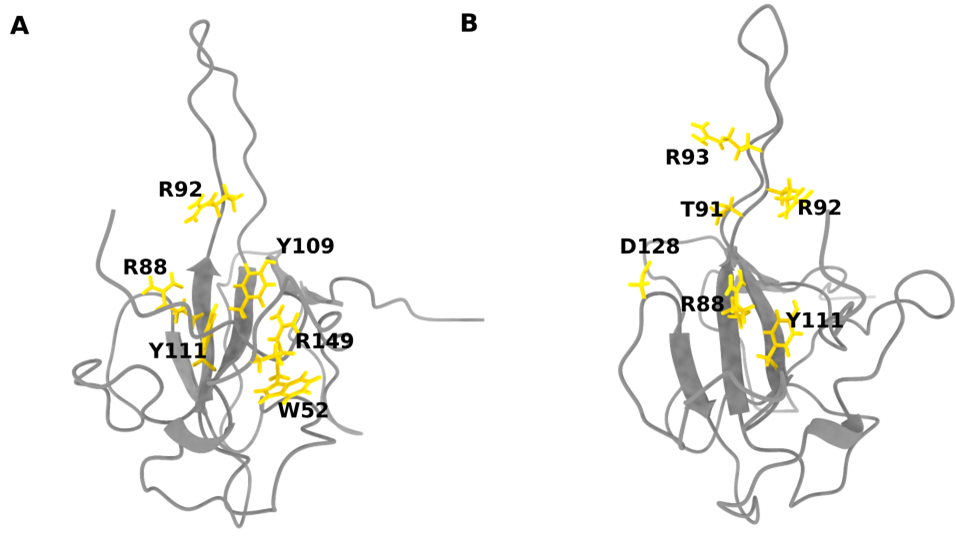


**Figure S18.** NTD(44–180) residues involved in intermolecular hydrogen bonds with peptides **P1** and **P2** during MD simulations, identified based on hydrogen bond occupancy (>20%) over the trajectories (cpptraj analysis). A) NTD(44–180) interactions with **P1**. B) NTD(44–180) interactions with **P2**. NTD(44–180) residues frequently participating in hydrogen bonds are highlighted in gold.

**Table S9 – Hydrogen bonds from peptide-protein interactions.** Hydrogen bonds were identified in representative structures from clusters obtained from MD trajectories. Measurements were performed in ChimeraX, and the table reports donor/acceptor atoms and corresponding geometric distances (D···A and D–H···A). The data show that for **P1**, the first cluster reveals several hydrogen bonds, primarily involving Arg residues (e.g., Arg93, Arg95, Arg92) interacting with acidic residues such as Glu1, Glu5, Glu14, Glu21, and Glu23. In addition to the interactions between the core of both NTD(44-180) and **P1**, simulations also show some contacts involving the remaining parts of the peptide, the most flexible ones (Lys100–Glu1, Arg149–Glu3, and polar contacts such as Tyr111–Gly4, and Ser51–Glu3). Notably, Arg93 forms multiple contacts with Glu21 and Glu23, suggesting a potential anchoring role. For **P2**, the first cluster exhibits a broader distribution of hydrogen bonds. Key residues such as Arg93, Arg95, and Lys127 form multiple hydrogen bonds with residues Glu13, Glu17, Glu20 and Glu4, while hydrophobic and backbone contacts such as Asn47–Leu16 and Thr91-Leu14 also emerge. The second cluster adds further contacts, notably involving Arg88, Arg93, Lys100 and Lys127, including recurrent interactions with Glu13 and Glu4, suggesting that these interactions may serve as interaction hotspots for this peptide.

| **Name** | **Donor** | **Donor Atom** | **Acceptor** | **Acceptor Atom** | **Hydrogen** | **Dist D..A** | **Dist D-H..A** |
| --- | --- | --- | --- | --- | --- | --- | --- |
| P1 Cluster 1 | SER51 | N | GLU3 | O | H | 2.948 | 1.977 |
|  | ARG88 | NH1 | GLY7 | O | HH11 | 2.725 | 1.75 |
|  | ARG92 | NE | GLU5 | OE1 | HE | 2.901 | 1.978 |
|  | ARG92 | NE | GLU5 | OE2 | HE | 3.228 | 2.374 |
|  | ARG92 | NH2 | GLU5 | OE1 | HH21 | 2.867 | 2.022 |
|  | ARG93 | NH1 | GLU21 | OE1 | HH11 | 3.309 | 2.434 |
|  | ARG93 | NH1 | GLU23 | OE2 | HH12 | 3.037 | 2.049 |
|  | ARG93 | NH2 | GLU23 | OE1 | HH22 | 2.852 | 1.846 |
|  | ILE94 | N | GLU21 | OE1 | H | 2.91 | 2.11 |
|  | ILE94 | N | GLU21 | OE2 | H | 2.786 | 1.824 |
|  | ARG95 | N | GLU21 | OE2 | H | 2.958 | 1.948 |
|  | ARG95 | NE | GLY20 | O | HE | 2.755 | 1.809 |
|  | ARG95 | NH1 | GLU14 | OE2 | HH12 | 2.865 | 1.927 |
|  | ARG95 | NH2 | GLU14 | OE1 | HH22 | 3.093 | 2.13 |
|  | ARG95 | NH2 | GLU14 | OE2 | HH22 | 3.058 | 2.301 |
|  | ARG95 | NH2 | GLY20 | O | HH21 | 2.993 | 2.262 |
|  | LYS100 | NZ | GLU1 | OE1 | HZ2 | 2.697 | 1.897 |
|  | TYR111 | OH | GLY4 | O | HH | 2.564 | 1.617 |
|  | ARG149 | NH1 | GLU3 | OE2 | HH12 | 2.969 | 2.039 |
|  | ARG149 | NH2 | GLU3 | OE2 | HH22 | 2.911 | 1.924 |
| P1 Cluster 2 | THR49 | OG1 | GLU5 | O | HG1 | 3.41 | 2.687 |
|  | SER51 | N | GLU3 | O | H | 2.948 | 1.977 |
|  | SER51 | N | GLU3 | O | H | 2.261 | 1.317 |
|  | ARG88 | NH1 | GLY7 | O | HH11 | 2.725 | 1.75 |
|  | ARG92 | NE | GLU5 | OE1 | HE | 2.901 | 1.978 |
|  | ARG92 | NE | GLU5 | OE2 | HE | 3.228 | 2.374 |
|  | ARG92 | NH2 | GLU5 | OE1 | HH21 | 2.867 | 2.022 |
|  | ARG93 | NH1 | GLU21 | OE1 | HH11 | 3.309 | 2.434 |
|  | ARG93 | NH1 | GLU23 | OE2 | HH12 | 3.037 | 2.049 |
|  | ARG93 | NH2 | GLU23 | OE1 | HH22 | 2.852 | 1.846 |
|  | ILE94 | N | GLU21 | OE1 | H | 2.91 | 2.11 |
|  | ILE94 | N | GLU21 | OE2 | H | 2.786 | 1.824 |
|  | ARG95 | N | GLU21 | OE2 | H | 2.958 | 1.948 |
|  | ARG95 | NE | GLY20 | O | HE | 2.755 | 1.809 |
|  | ARG95 | NH1 | GLU14 | OE2 | HH12 | 2.865 | 1.927 |
|  | ARG95 | NH2 | GLU14 | OE1 | HH22 | 3.093 | 2.13 |
|  | ARG95 | NH2 | GLU14 | OE2 | HH22 | 3.058 | 2.301 |
|  | ARG95 | NH2 | GLY20 | O | HH21 | 2.993 | 2.262 |
|  | LYS100 | NZ | GLU1 | OE1 | HZ2 | 2.697 | 1.897 |
|  | TYR111 | OH | GLY4 | O | HH | 2.564 | 1.617 |
|  | TYR111 | OH | GLY4 | O | HH | 2.228 | 1.397 |
|  | ARG149 | NH1 | GLU3 | OE2 | HH12 | 2.969 | 2.039 |
|  | ARG149 | NH1 | GLU3 | OE2 | HH12 | 3.028 | 2.091 |
|  | ARG149 | NH2 | GLU3 | OE2 | HH22 | 2.911 | 1.924 |
|  | ARG149 | NH2 | GLU3 | OE2 | HH22 | 2.949 | 1.968 |
|  | SER51 | N | GLU3 | O | H | 2.956 | 2.068 |
|  | ARG93 | NE | GLU14 | OE1 | HE | 2.922 | 2.07 |
|  | ARG93 | NH2 | GLU14 | OE1 | HH21 | 2.839 | 1.908 |
|  | ARG95 | NH1 | GLU14 | OE1 | HH11 | 3.375 | 2.66 |
|  | ARG95 | NH1 | GLU14 | OE2 | HH11 | 3.084 | 2.173 |
|  | LYS100 | NZ | GLU21 | O | HZ1 | 3.009 | 2.04 |
|  | LYS100 | NZ | GLU21 | OE1 | HZ2 | 2.806 | 1.806 |
|  | ARG107 | NH1 | GLU1 | OE1 | HH12 | 2.811 | 1.845 |
|  | ARG107 | NH2 | GLU1 | O | HH22 | 2.729 | 1.84 |
|  | TYR111 | OH | GLY4 | O | HH | 2.833 | 1.878 |
|  | ARG149 | NH1 | GLU3 | OE2 | HH12 | 3.298 | 2.501 |
|  | ARG149 | NH1 | GLU3 | OE2 | HH12 | 3.401 | 2.637 |
|  | ARG149 | NH2 | GLU3 | OE2 | HH22 | 2.831 | 1.891 |
|  | ARG149 | NH2 | GLU3 | OE2 | HH22 | 2.867 | 1.9 |
| P2 Cluster 1 | ASN47 | ND2 | LEU16 | O | HD22 | 3.023 | 2.051 |
|  | ARG88 | NH2 | GLU13 | OE1 | HH21 | 3.353 | 2.376 |
|  | ARG88 | NH2 | GLU13 | OE2 | HH21 | 2.986 | 2.151 |
|  | THR91 | N | GLU13 | O | H | 2.794 | 1.82 |
|  | THR91 | OG1 | GLU13 | O | HG1 | 3.005 | 2.108 |
|  | ARG93 | N | LEU14 | O | H | 2.821 | 1.864 |
|  | ARG93 | NE | LEU19 | O | HE | 3.166 | 2.38 |
|  | ARG93 | NH1 | GLY15 | O | HH12 | 3.245 | 2.374 |
|  | ARG93 | NH1 | GLU17 | OE2 | HH11 | 3 | 2.176 |
|  | ARG95 | NH1 | GLU20 | OE1 | HH12 | 2.972 | 2.034 |
|  | ARG95 | NH2 | GLU20 | OE1 | HH22 | 2.902 | 1.957 |
|  | LYS100 | NZ | GLU4 | OE2 | HZ3 | 3.03 | 2.185 |
|  | TYR111 | OH | GLU13 | OE1 | HH | 2.562 | 1.62 |
|  | LYS127 | NZ | GLU4 | O | HZ3 | 2.821 | 1.919 |
|  | ASP128 | N | LEU3 | O | H | 2.966 | 1.987 |
|  | LEU14 | N | THR91 | O | H | 2.666 | 1.805 |
| P2 Cluster 2 | ASN47 | ND2 | LEU16 | O | HD22 | 3.023 | 2.051 |
|  | ARG88 | NE | GLU13 | OE2 | HE | 3.205 | 2.416 |
|  | ARG88 | NH2 | GLU13 | OE1 | HH21 | 3.353 | 2.376 |
|  | ARG88 | NH2 | GLU13 | OE2 | HH21 | 2.986 | 2.151 |
|  | ARG88 | NH2 | GLU13 | OE1 | HH21 | 2.888 | 2.074 |
|  | ARG88 | NH2 | GLU13 | OE2 | HH21 | 2.968 | 2.071 |
|  | THR91 | N | GLU13 | O | H | 2.794 | 1.82 |
|  | THR91 | N | GLU13 | O | H | 2.465 | 1.464 |
|  | THR91 | OG1 | GLU13 | O | HG1 | 3.005 | 2.108 |
|  | THR91 | OG1 | GLU13 | O | HG1 | 2.773 | 1.841 |
|  | ARG93 | N | LEU14 | O | H | 2.821 | 1.864 |
|  | ARG93 | N | LEU14 | O | H | 2.259 | 1.383 |
|  | ARG93 | NE | LEU19 | O | HE | 3.166 | 2.38 |
|  | ARG93 | NE | GLU22 | OE1 | HE | 3.421 | 2.461 |
|  | ARG93 | NH1 | GLY15 | O | HH12 | 3.245 | 2.374 |
|  | ARG93 | NH1 | GLU17 | OE2 | HH11 | 3 | 2.176 |
|  | ARG95 | NH1 | GLU20 | OE1 | HH12 | 2.972 | 2.034 |
|  | ARG95 | NH2 | GLU20 | OE1 | HH22 | 2.902 | 1.957 |
|  | LYS100 | NZ | GLU4 | OE2 | HZ3 | 3.03 | 2.185 |
|  | LYS100 | NZ | GLU4 | OE2 | HZ3 | 3.177 | 2.244 |
|  | TYR111 | OH | GLU13 | OE1 | HH | 2.562 | 1.62 |
|  | TYR111 | OH | GLU13 | OE2 | HH | 3.243 | 2.325 |
|  | LYS127 | NZ | GLU4 | O | HZ3 | 2.821 | 1.919 |
|  | ASP128 | N | LEU3 | O | H | 2.966 | 1.987 |
|  | LEU14 | N | THR91 | O | H | 2.666 | 1.805 |
|  | ASN47 | ND2 | LEU16 | O | HD22 | 2.923 | 1.913 |
|  | ARG88 | NE | GLU13 | OE2 | HE | 2.765 | 1.785 |
|  | ARG88 | NH2 | GLU13 | OE1 | HH21 | 3.518 | 2.638 |
|  | ARG88 | NH2 | GLU13 | OE2 | HH21 | 3.046 | 2.083 |
|  | ARG88 | NH2 | GLU13 | OE1 | HH21 | 2.93 | 1.978 |
|  | ARG88 | NH2 | GLU13 | OE2 | HH21 | 2.963 | 2.173 |
|  | THR91 | N | GLU13 | O | H | 3.465 | 2.559 |
|  | THR91 | N | GLU13 | O | H | 3.119 | 2.268 |
|  | THR91 | OG1 | GLU13 | O | HG1 | 3.491 | 2.674 |
|  | ARG93 | N | LEU14 | O | H | 2.977 | 2.002 |
|  | ARG93 | NH1 | GLU17 | O | HH12 | 2.215 | 1.352 |
|  | ARG93 | NH1 | GLY11 | O | HH11 | 3.4 | 2.481 |
|  | ARG93 | NH1 | GLY18 | O | HH12 | 3.049 | 2.105 |
|  | ARG93 | NH2 | GLU17 | O | HH22 | 2.923 | 2.074 |
|  | LYS100 | NZ | GLU4 | OE2 | HZ2 | 2.701 | 1.801 |
|  | LYS100 | NZ | GLU4 | OE1 | HZ2 | 3.016 | 2.243 |
|  | LYS100 | NZ | GLU4 | OE2 | HZ2 | 2.778 | 1.87 |
|  | TYR111 | OH | GLU13 | OE1 | HH | 1.904 | 1.023 |
|  | TYR111 | OH | GLU13 | OE2 | HH | 2.615 | 1.669 |
|  | LYS127 | NZ | GLU2 | OE1 | HZ2 | 2.927 | 1.931 |
|  | LYS127 | NZ | GLU4 | O | HZ1 | 2.975 | 2.327 |
|  | LYS127 | NZ | GLU7 | O | HZ1 | 3.217 | 2.284 |
|  | ASP128 | N | LEU3 | O | H | 2.945 | 1.994 |
|  | LEU14 | N | THR91 | O | H | 2.162 | 1.389 |
|  | LEU14 | N | THR91 | O | H | 2.977 | 1.989 |
|  | GLY18 | N | PRO46 | O | H | 2.925 | 1.987 |

**Table S10.** **High-frequency salt bridges at the protein-peptide interface (>50%).** The table reports the peptide-protein salt bridges (contacts below 3.2Å). Our analysis identified several high-occupancy salt bridges (>50% of the trajectory) at the peptide-NTD(44-180) interface. For **P1**, the most persistent salt bridges predominantly involve peptide glutamate residues interacting with arginine and lysine NTD(44-180) residues, including Glu14–Arg88, Glu14–Arg92, Glu1–Arg149, and Glu19–Lys127. Similarly, **P2** displays a consistent pattern of salt bridges formation, involving Glu13–Arg88, Glu17–Arg92, Glu13–Arg149, and Glu2–Lys127. The recurrence of Arg88, Arg92 and Arg149 across both peptides highlights these residues as key electrostatic hotspots at the NTD(44-180) surface. Together, the results obtained from the data reported in Table S9 and S10 indicate that salt bridges complement the hydrogen-bond network by providing electrostatic stabilization to the peptide ligands.

| **Peptide** | **Residue Pairs (Peptide-Protein)** |
| --- | --- |
| P1 | GLU14-ARG88 |
|  | GLU3-ARG149 |
|  | GLU14-ARG92 |
|  | GLU1-ARG149 |
|  | GLU10-ARG92 |
|  | GLU14-ARG93 |
|  | GLU10-ARG88 |
|  | GLU5-ARG89 |
|  | GLU21-ARG92 |
|  | GLU1-ARG95 |
|  | GLU14-ARG95 |
|  | GLU1-ARG92 |
|  | GLU19-LYS127 |
| P2 | GLU13-ARG88 |
|  | GLU7-ARG92 |
|  | GLU10-ARG88 |
|  | GLU17-ARG92 |
|  | GLU13-ARG149 |
|  | GLU2-LYS127 |
|  | GLU4-ARG95 |
|  | GLU10-ARG93 |
|  | GLU7-ARG88 |

**NMR Experiments**

**Table S11.** Acquisition parameters of NMR experiments for peptides structural characterization and protein-ligand interaction

| **Construct** | **Experiment** | **Data points** | | **Spectral width (Hz)** | | **Number of Scans** | **Interscan delay (s)** |
| --- | --- | --- | --- | --- | --- | --- | --- |
|  |  | **F2** | **F1** | **F2** | **F1** |  |  |
| **P1/P2** | 2D ^1^H-^1^H TOCSY | 32768 | 1800 | 9090 | 9009 | 4 | 4.8 |
| **P1/P2** | 2D ^1^H-^1^H NOESY | 32768 | 1800 | 9090 | 9009 | 8 | 4.8 |
| **P1/P2** | 2D ^1^H-^15^N HSQC^4^ | 2048 | 400 | 13158 | 2375 | 72 | 2.08 |
| **P1/P2** | 2D ^1^H-^13^C HSQC^5^ | 2048 | 512 | 14705 | 15151 | 48 | 2.07 |
| NTD(44-180)+**P1/P2** | 2D ^1^H-^15^N HSQC^4^ | 8192 | 256 | 10869 | 2432 | 16 | 1.10 |
| NTR(1-248) + **P0** | 2D ^1^H-^15^N HSQC^4^ | 4096 | 640 | 19230 | 3472 | 8 | 1.10 |

**Table S12.** Acquisition parameters of NMR experiments for peptides dynamic properties characterization

| **Construct** | **Experiment** | **Data points** | **Spectral width (Hz)** | **Number of Scans** | **Interscan delays (s)** |
| --- | --- | --- | --- | --- | --- |
| **P1/P2** | 1D | 65536 | 22727 | 32 | 2.54 |
| **P1/P2** | CPMG | 65536 | 22727 | 64 | 6.44 |
| **P1/P2** | I.R. | 65536 | 22727 | 64 | 6.44 |
| NTD(44-180) | 1D | 65536 | 22727 | 32 | 2.54 |
| NTD(44-180) | CPMG | 65536 | 22727 | 32 | 6.44 |
| NTD(44-180) | I.R. | 65536 | 22727 | 64 | 6.44 |

**References**

1. Piovesan, D., Walsh, I., Minervini, G. & Tosatto, S. C. E. FELLS: fast estimator of latent local structure. *Bioinformatics* **33**, 1889–1891 (2017).

2. Heller, G. T., Shukla, V. K., Figueiredo, A. M. & Hansen, D. F. Picosecond Dynamics of a Small Molecule in Its Bound State with an Intrinsically Disordered Protein. *J. Am. Chem. Soc.* **146**, 2319–2324 (2024).

3. Overbeck, J. H., Kremer, W. & Sprangers, R. A suite of 19F based relaxation dispersion experiments to assess biomolecular motions. *J. Biomol. NMR* **74**, 753–766 (2020).

4. Palmer, A. G., Cavanagh, J., Wright, P. E. & Rance, M. Sensitivity improvement in proton-detected two-dimensional heteronuclear correlation NMR spectroscopy. *J. Magn. Reson. 1969* **93**, 151–170 (1991).

5. Schleucher, J. *et al.* A general enhancement scheme in heteronuclear multidimensional NMR employing pulsed field gradients. *J. Biomol. NMR* **4**, (1994).
